# Integrated genomic characterization of oesophageal carcinoma

**DOI:** 10.1038/nature20805

**Published:** 2017-01-04

**Authors:** The Cancer Genome Atlas Research Network

## Abstract

Oesophageal cancers are prominent worldwide; however, there are few targeted therapies and survival rates for these cancers remain dismal. Here we performed a comprehensive molecular analysis of 164 carcinomas of the oesophagus derived from Western and Eastern populations. Beyond known histopathological and epidemiologic distinctions, molecular features differentiated oesophageal squamous cell carcinomas from oesophageal adenocarcinomas. Oesophageal squamous cell carcinomas resembled squamous carcinomas of other organs more than they did oesophageal adenocarcinomas. Our analyses identified three molecular subclasses of oesophageal squamous cell carcinomas, but none showed evidence for an aetiological role of human papillomavirus. Squamous cell carcinomas showed frequent genomic amplifications of *CCND1* and *SOX2* and/or *TP63*, whereas *ERBB2, VEGFA* and *GATA4* and *GATA6* were more commonly amplified in adenocarcinomas. Oesophageal adenocarcinomas strongly resembled the chromosomally unstable variant of gastric adenocarcinoma, suggesting that these cancers could be considered a single disease entity. However, some molecular features, including DNA hypermethylation, occurred disproportionally in oesophageal adenocarcinomas. These data provide a framework to facilitate more rational categorization of these tumours and a foundation for new therapies.

Oesophageal cancers have 5-year survival rates of 12–20% in Western populations^1,2^ and cause the deaths of over 400,000 people worldwide annually^3^. Oesophageal cancer is classified by histology as adenocarcinoma (EAC) or squamous cell carcinoma (ESCC)^4^. EAC incidence has increased several fold in Western countries in recent decades^5^, occurs predominantly in the lower oesophagus near the gastric junction, and is associated with obesity, gastric reflux and a precursor state termed Barrett’s oesophagus. Rising EAC rates are paralleled by increasing incidences of proximal stomach cancer^6^. ESCCs predominate in the upper and mid-oesophagus and are associated with smoking and alcohol exposure in Western populations. In non-Western countries, risk factors for ESCCs are less established.

The appropriate demarcation between gastric and oesophageal adenocarcinomas and the classification of adenocarcinomas spanning the gastroesophageal junction (GEJ) remain unresolved^7–9^, and there is debate regarding the utility of histological distinctions^4^. To improve oesophageal cancer classification, we performed a comprehensive molecular analysis of 164 oesophageal tumours, 359 gastric adenocarcinomas and 36 additional adenocarcinomas at the GEJ. We evaluated approaches for categorizing oesophageal tumours and identified molecular features and candidate pathways that define molecular subgroups and offer potential therapeutic targets.

## Sample collection and molecular characterization

**Figure 1:**
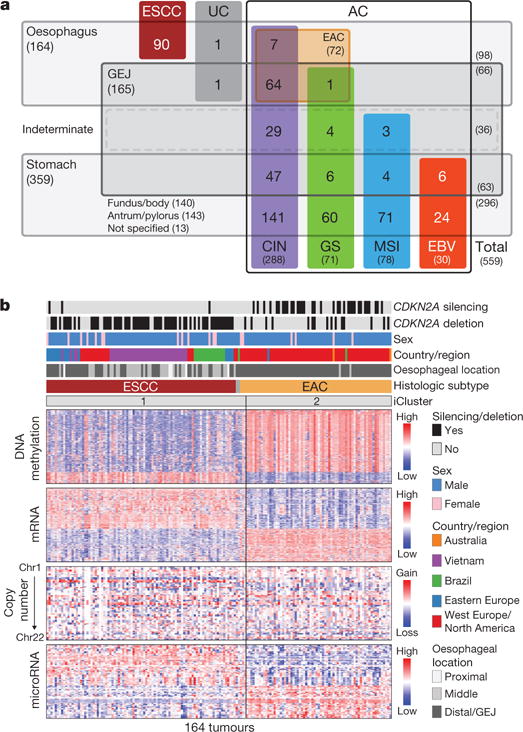
Major subdivisions of gastroesophageal cancer **a**, 559 oesophageal and gastric carcinoma tumours were categorized into sample sets. CIN, chromosomal instability; EBV, Epstein–Barr virus; GEJ, gastroesophageal junction; GS, genomically stable; MSI, microsatellite instability. UC, undifferentiated carcinoma. **b**, Integrated clustering of four molecular platforms shows that oesophageal carcinomas fall into two molecular subtypes (iCluster 1 and iCluster 2) that are virtually identical to histological classes ESCC and EAC. Clinical (top) and molecular data (bottom) from 164 tumours profiled with all four platforms are depicted.

We addressed the challenge of clinically distinguishing oesophageal and gastric adenocarcinomas through review of adenocarcinomas originating near the GEJ, using anatomic data and histopathologic criteria, to categorize tumours by oesophageal, gastric or indeterminate origins (Fig. 1a, Supplementary Table 1, Supplementary Fig. 1.1). We identified 90 ESCCs, 72 EACs (61 definite oesophageal and 11 probable oesophageal), 36 GEJ carcinomas of indeterminate origin, 63 gastric GEJ carcinomas (15 definite gastric and 48 probable gastric), 140 gastric carcinomas of the fundus or body, and 143 gastric antral or pyloric carcinomas. We were unable to localize 13 gastric adenocarcinomas more narrowly within the stomach, and 2 oesophageal tumours were undifferentiated carcinomas.

Fresh-frozen tumour samples from patients who were not previously treated with chemotherapy or radiation therapy were obtained from multiple countries with informed consent and local Institutional Review Board approval. Germline DNA was collected from blood or nonmalignant oesophageal mucosa. Genetic material was subjected to whole-exome sequencing, single-nucleotide polymorphism (SNP) array profiling to evaluate somatic copy-number alterations (SCNAs), DNA methylation profiling and mRNA and microRNA sequencing. DNA from 51 oesophageal cancers was subjected to low-pass (6–8 × coverage) whole-genome sequencing. Reverse-phase protein array proteomic analysis was performed on 113 tumours.

## Molecular separation of ESCC and EAC

**Extended Data Figure 1:**
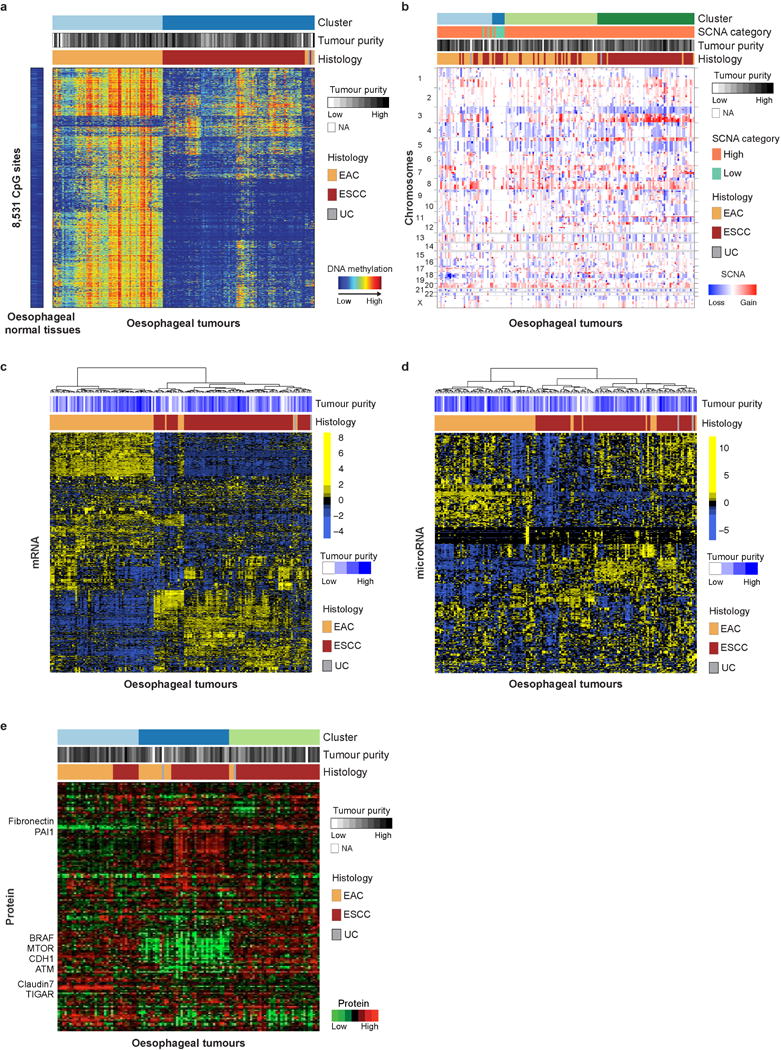
Platform-specific unsupervised clustering analyses of oesophageal cancers **a–e**, Unsupervised clustering of oesophageal cancers based on DNA hypermethylation **(a)**, SCNAs **(b)**, gene expression profiles **(c)**, microRNA profiles **(d)** and reverse-phase protein array data **(e)** revealed strong separation between EAC and ESCC in multiple molecular platforms. Samples are displayed as columns. EAC, oesophageal adenocarcinoma; ESCC, oesophageal squamous cell carcinoma; UC, undifferentiated carcinoma.

**Extended Data Figure 2:**
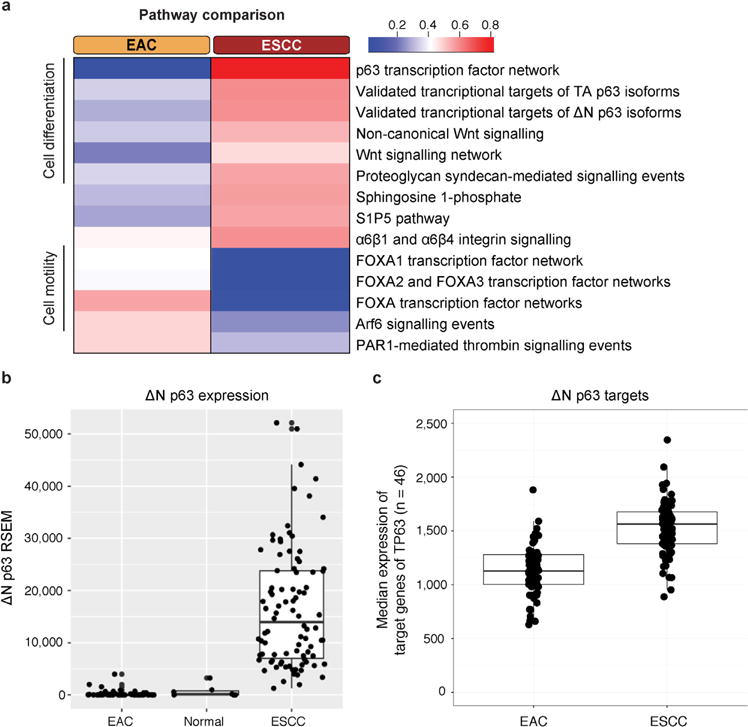
Pathways with significant expression differences between EAC and ESCC **a**, NCI PID pathways in which expression differs significantly between EAC and ESCC (*P_s_* < 10^−3^, where *P_s_* is the statistical significance of the pathway score (see Methods)) are listed. The colour scale shows the median (log_2_) expression value of significantly differentially expressed genes (*P* < 10^−3^) in the corresponding pathway, normalized to unit range. **b**, TP63ΔN transcript levels were measured in EAC, solid tissue normal, and ESCC samples. **c**, Median gene expression values of genes in the NCI-PID pathway ‘Validated transcriptional targets of the ΔN p63 isoforms’ in EAC and ESCC. Each point represents one sample, and the value is the median expression value of the 46 genes in the pathway.

We evaluated the 164 oesophageal carcinomas using integrated clustering of SCNA, DNA methylation, mRNA and microRNA expression data using iCluster10. Both independent and integrated analyses from each molecular platform revealed separation between squamous cancers and adenocarcinomas (Fig. 1b; Extended Data Fig. 1 a–e). Gene expression analysis (Extended Data Fig. 2) revealed that EACs showed increased E-cadherin (*CDH1*) signalling and upregulation of ARF6 and FOXA pathways, which regulate E-cadherin11. By contrast, ESCCs exhibited upregulation of Wnt, syndecan and p63 pathways, the latter being essential for squamous epithelial cell differentiation12. These data suggest the presence of lineage-specific alterations that drive progression in EACs and ESCCs.

## Somatic genomic alterations in oesophageal cancer

**Extended Data Figure 3:**
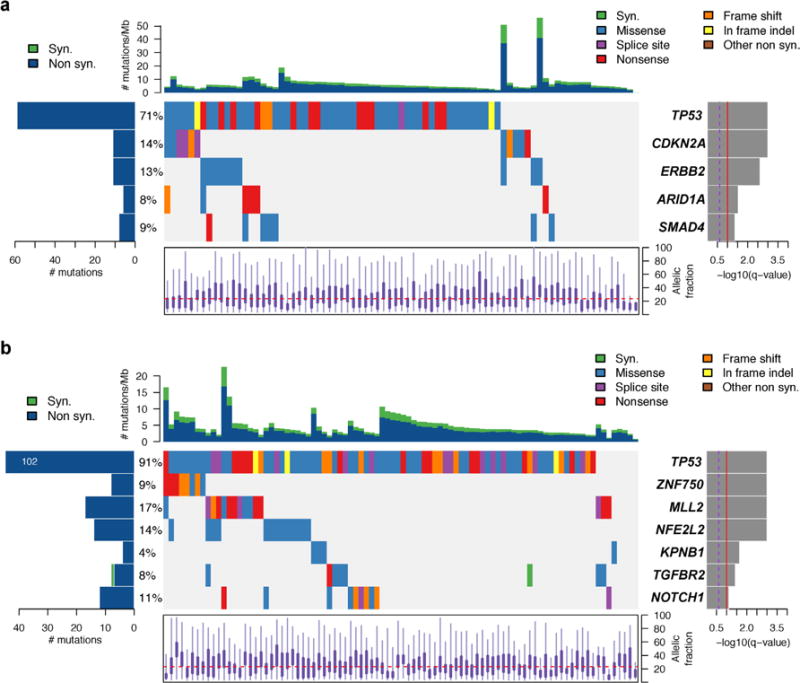
MutSig analyses of significantly mutated genes in EAC and ESCC **a**, Plot of significantly mutated genes from the MutSigCV2 computational analysis of whole-exome sequencing data from EAC samples. Genes are ordered by level of significance (*q* value as plotted at right). At left is the prevalence of each mutation in the sample set. The coloured boxes show samples with specific mutations, with the type of mutation labelled by box colour, with legend at upper right. The top plot shows the number of mutations per sample with synonymous (Syn.) and non-synonymous (Non syn.) mutations plotted separately. The bottom plot shows the distribution of allelic fraction of mutations for the samples sequenced. **b**, The MutSig plot for ESCC is shown the same as for the EAC samples above.

**Extended Data Figure 4:**
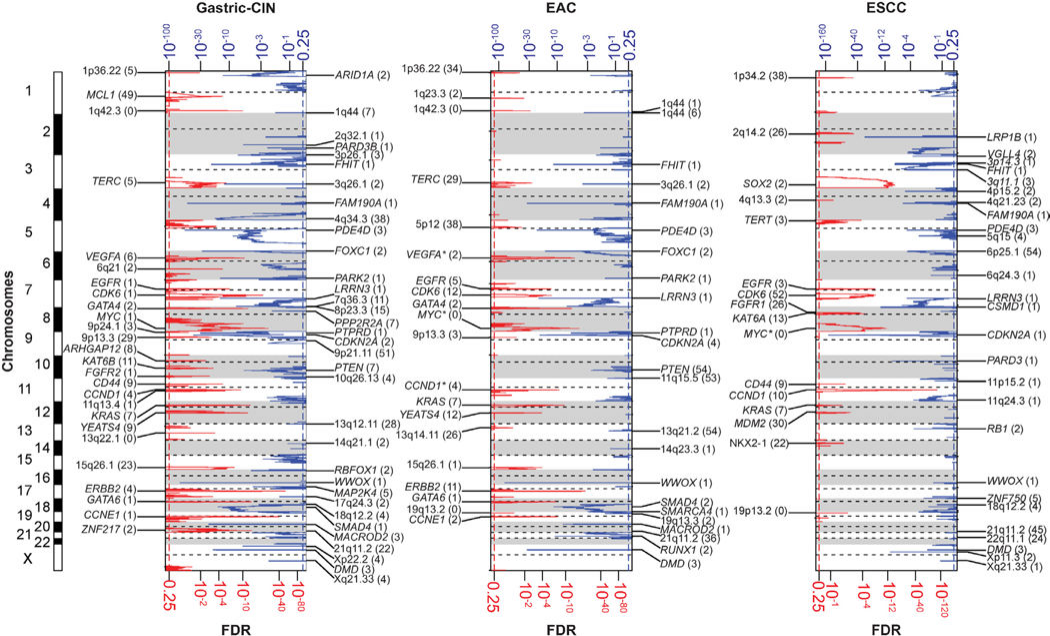
GISTIC analysis of foci of recurrent amplification and deletion These figures demonstrate foci of significantly recurrent focal amplification and deletion as determined from GISTIC 2.0 analysis of somatic copy number data from SNP arrays. Separate plots are shown for CIN-gastric cancer (left), EAC (middle) and ESCC (right). Each plot arrays the chromosomes from 1 (top) to X (bottom) and shows foci of significant amplification (left, red with scale at bottom) or deletion (right, blue with scale at top). Candidate targets of each focus of amplification or deletion are shown in the label for the respective peak. Peaks without clear targets are labelled by chromosome band. The number in parentheses indicates the number of genes in each peak as calculated by GISTIC. Genes marked with asterisks are likely drivers located adjacent to peak areas defined by GISTIC.

We evaluated somatic genomic alterations separately in ESCC and EAC using MutSig13 to search for genes with significantly recurring mutations (Extended Data Fig. 3a, b). In ESCC, we identified significantly mutated genes, TP53, NFE2L2, MLL2, ZNF750, NOTCH1 and TGFBR2, consistent with previous studies14–20. In EAC, we identified significant mutations in TP53, CDKN2A, ARID1A, SMAD4 and ERBB2, as reported previously21. These findings are consistent with the prominence of CDKN2A and TP53 mutations in dysplastic Barrett’s oesophagus, a precursor to EAC. Similarly, we analysed SCNA data with GISTIC22 to define recurrently amplified and deleted regions (Extended Data Fig. 4; Supplementary Table 2). Although EAC and ESCC shared some recurring SCNAs, we confirmed substantial differences in patterns of alterations between the diseases19,23. SCNAs that were recurrent in EAC (but absent in ESCC) included amplifications containing VEGFA (6p21.1), ERBB2 (17p12), GATA6 (18q11.2) and CCNE1 (19q12), and deletion of SMAD4 (18q21.2). Recurring focal SCNAs in ESCC included amplifications of SOX2 (3q26.33), TERT (5p15.33), FGFR1 (8p11.23), MDM2 (12q14.3), NKX2-1 (14q13.2) and deletion of RB1 (13q14.2). We found novel focal deletions at 3p25.2 in ESCC, encompassing the negative regulator of the Hippo pathway VGLL4 and autophagy factor ATG7.

**Figure 2:**
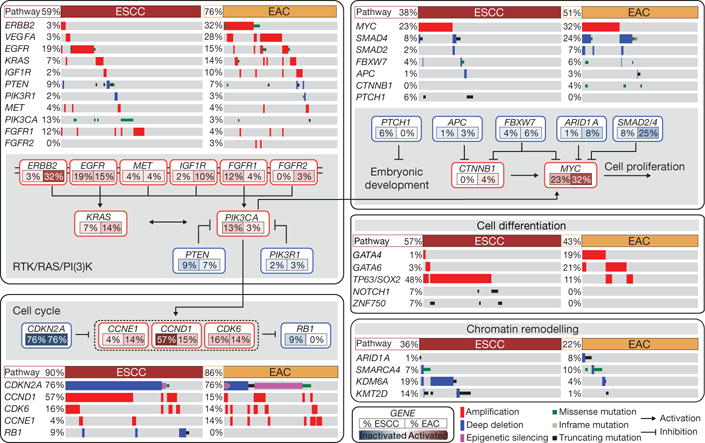
Integrated molecular comparison of somatic alterations across oesophageal cancer Mutations and SCNAs for selected genes and *CDKN2A* epigenetic silencing are shown for EACs and ESCCs. Genes are grouped by pathways, with lines and arrows showing pairwise molecular interactions. Deep deletions indicate loss of more than half of gene copies. Only missense mutations reported in the COSMIC repository are included. Alteration frequencies for each gene are listed inside rounded rectangles with ESCC rates on left and EAC on right, with red shading denoting gene activation, and blue denoting inactivation.

Combined mutation and SCNA data revealed frequent alterations in cell cycle regulators (Fig. 2). Inactivation of CDKN2A and amplification of CCND1 were present in 76% and 57% of squamous tumours, respectively; and additional ESCCs had amplification of CDK6 or loss of RB1. Patterns of cell-cycle dysregulation differed in EACs, where CCND1 was amplified in only 15% of tumours, but we observed more common amplification of CCNE1. CDKN2A was inactivated in 76% of EACs by mutation, deletion or epigenetic silencing. These data reveal a potential role for inhibitors of cell cycle kinases for treatment, especially in ESCC.

We found frequent alterations of receptor tyrosine kinases and downstream signalling mediators, particularly in EAC. In ESCCs, we identified amplification or mutation of EGFR in 19% of tumours and alterations of PIK3CA, PTEN or PIK3R1, all of which are believed to activate the PI3K pathway, in 24% of tumours. EACs had a wider range of potentially oncogenic amplifications, most commonly of ERBB2, which was altered in 32% of EACs, but in only 3% of ESCCs. Although clinical trials that led to approval by the US Food and Drug Administration of the ERBB2-directed antibody trastuzumab were limited to gastric and GEJ adenocarcinomas24, ERBB2-positive EACs are routinely treated off-label with trastuzumab. Notably, we found mutations of ERBB2 in four tumours lacking ERBB2 amplification, suggesting that more patients may benefit from ERBB2-directed therapy. Transcriptome data identified six cases with ERBB2 amplification that expressed a fusion transcript in which exon 12 of ERBB2 was fused to the 3′ untranslated region of neighbouring gene JUP (Supplementary Fig. 3.1; Supplementary Table 3). Because this fusion transcript omits the ERBB2 transmembrane and tyrosine kinase domains, its potential functionality is unclear. Other EACs showed amplification of KRAS, EGFR, IGF1R or VEGFA.

Additional analysis identified dysregulation of the TGF-β pathway and less frequent CTNNB1 (β-catenin) activation, both more common in EAC than ESCC. We found that 6% of ESCCs (but no EACs) had inactivating alterations of PTCH1, as previously described15, suggesting activated hedgehog signalling. ESCC tumours, like other squamous cancers, had amplifications of chromosome 3q, focused on the SOX2 locus25. Genes that encode SOX2 or squamous transcription factor p63, also on chromosome 3p, were amplified in 48% of ESCCs. Moreover, mutations in ZNF750 and NOTCH1 in ESCCs may similarly modulate squamous cell maturation15–20. In EACs, however, we found frequent amplifications of genes that encode GATA4 and GATA6 developmental factors, as described in gastric adenocarcinomas26,27 and (for GATA6), experimentally validated in EAC28.

Both EAC and ESCCs showed alterations of chromatin-modifying enzymes (Supplementary Fig. 3.2). Alterations affecting SWI/SNF-encoding genes ARID1A, SMARCA4 and PBRM1 were more common in adenocarcinomas, whereas ESCCs contained more frequent alterations in histone-modifying factors KDM6A (UTX), KMT2D (MLL2) and KMT2C (MLL3). Therefore, although many of the same pathways were somatically altered in EACs and ESCCs, the specific genes affected were dissimilar, probably reflecting distinct pathophysiology and suggesting different therapeutic approaches. These data caution against performing clinical trials in mixed populations of EACs and ESCCs.

## Molecular subtypes of oesophageal SCC

**Figure 3:**
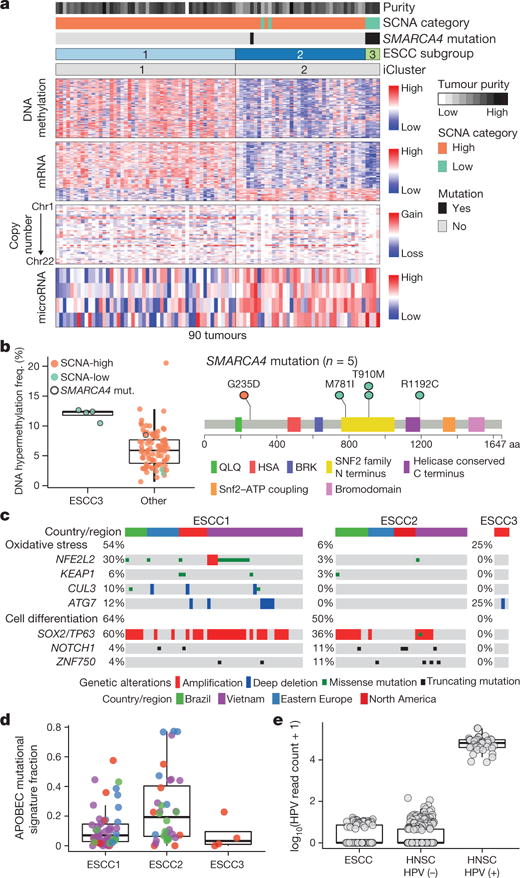
Distinct molecular subtypes of oesophageal squamous cell carcinoma **a**, ESCCs separated into subtypes ESCC1 and ESCC2 by iCluster, with identification of an additional group ESCC3 having *SMARCA4* mutations and reduced SCNAs. Clinical and molecular features are listed at top with molecular data at bottom. **b**, Left, DNA hypermethylation in ESCC3 and other ESCCs. Right, *SMARCA4* mutations. **c**, Genomic alterations that affect oxidative stress and cell differentiation in ESCC subtypes with samples segregated by geographic origin. **d**, Fraction of mutations with APOBEC signature by subtype and geographic origin. **e**, Human papilloma virus (HPV) transcript levels in oesophageal and head and neck SCCs.

Integrative clustering of ESCC data using iCluster revealed two classes, denoted iCluster 1 and iCluster 2 (Fig. 3a). Within iCluster 2, we identified a group of tumours with shared features including mutations in SMARCA4 (encoding the SWI/SNF factor BRG1), increased DNA methylation (Fig. 3a, rightmost samples) and relatively unaltered SCNA profiles (Fig. 3b). We designated the distinct set of tumours with these features as subtype ESCC3, thus dividing ESCCs into three molecular subtypes: ESCC1 (n = 50), ESCC2 (n = 36) and ESCC3 (n = 4).

**Extended Data Figure 5:**
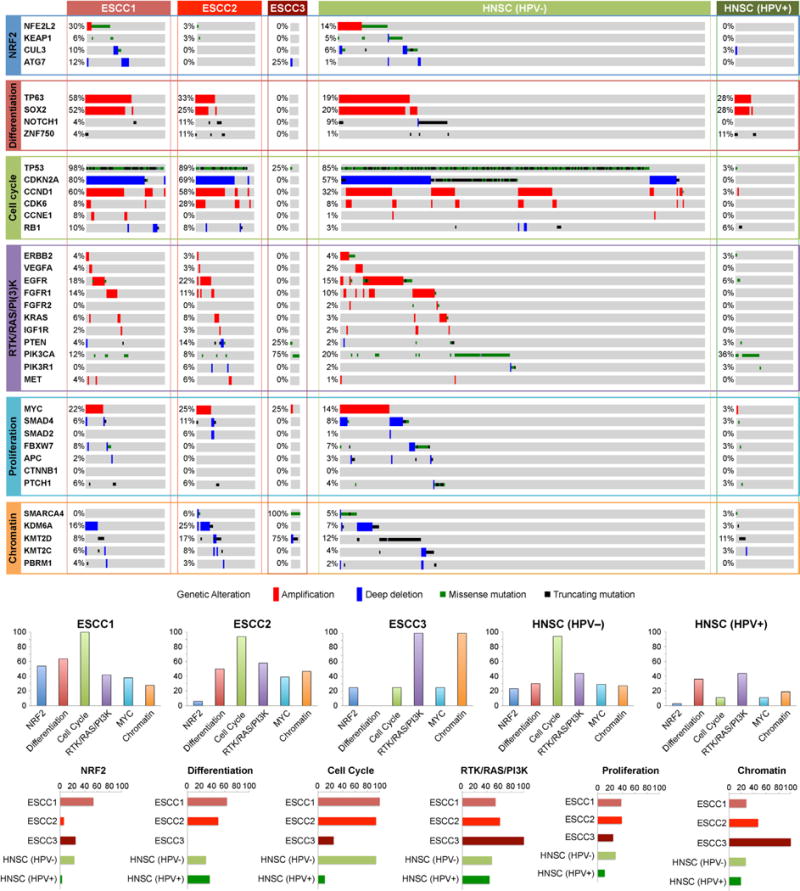
Comparison of somatic alterations in ESCC and HNSC subtypes Mutations and copy-number changes for selected genes in selected signalling pathways are shown for the three ESCC subtypes identified in our study and the HPV-negative (*n* = 243) and HPV-positive (*n* = 36) subtypes that had previously been identified by TCGA in the HNSC study. Amplifications and deep deletions indicate a change of more than half of the baseline gene copies. Missense mutations were included if they were found in the COSMIC repository. Alteration frequencies are expressed as percentage of altered cases within each molecular subtype. Bottom panels show percentage of altered cases per signalling pathway for each molecular subtype and percentage of altered cases per molecular subtype for each signalling pathway.

**Extended Data Figure 6:**
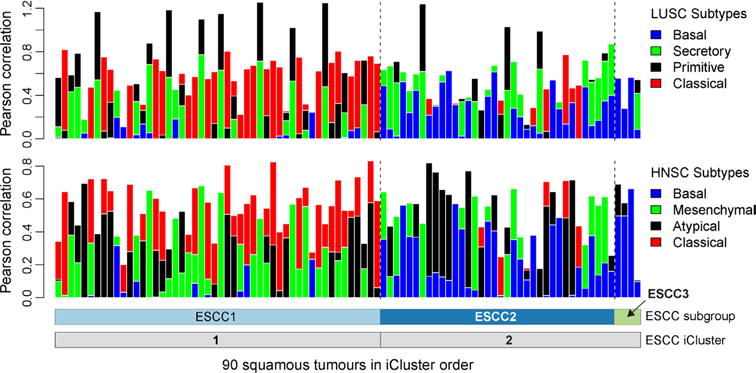
Distinct clusters of ESCC Columns indicate Pearson correlation between each of the mRNA profiles of 90 ESCC tumours with the centroids of the mRNA expression profiling subtypes that were developed for lung squamous cell carcinoma (LUSC, top) and head and neck squamous cell carcinoma (HNSC, bottom) gene expression analyses. Samples are in ESCC cluster order as in Fig. 3a.

ESCC1 was characterized by alterations in the NRF2 pathway, which regulates adaptation to oxidative stressors including some carcinogens and some chemotherapy agents. Mutations in NFE2L2 (NRF2), are associated with poor prognosis and resistance to chemoradiotherapy29. Alterations were seen in NFE2L2, in genes encoding proteins that degrade NRF2 (KEAP1 and CUL3), and in ATG7, encoding an NRF2 pathway autophagy factor30,31 (Fig. 3c). ESCC1 had a higher frequency of SOX2 and/or TP63 amplification (Fig. 3c, Extended Data Fig. 5). ESCC1 gene expression resembled the classical subtype described in The Cancer Genome Atlas (TCGA) studies of lung SCC32 and head and neck SCC (HNSCC)33 (Extended Data Fig. 6), which possess similar somatic alterations. ESCC1 showed higher rates of YAP1 (11q22.1) amplification and VGLL4/ATG7 deletion, suggesting activation of Hippo.

**Extended Data Figure 7:**
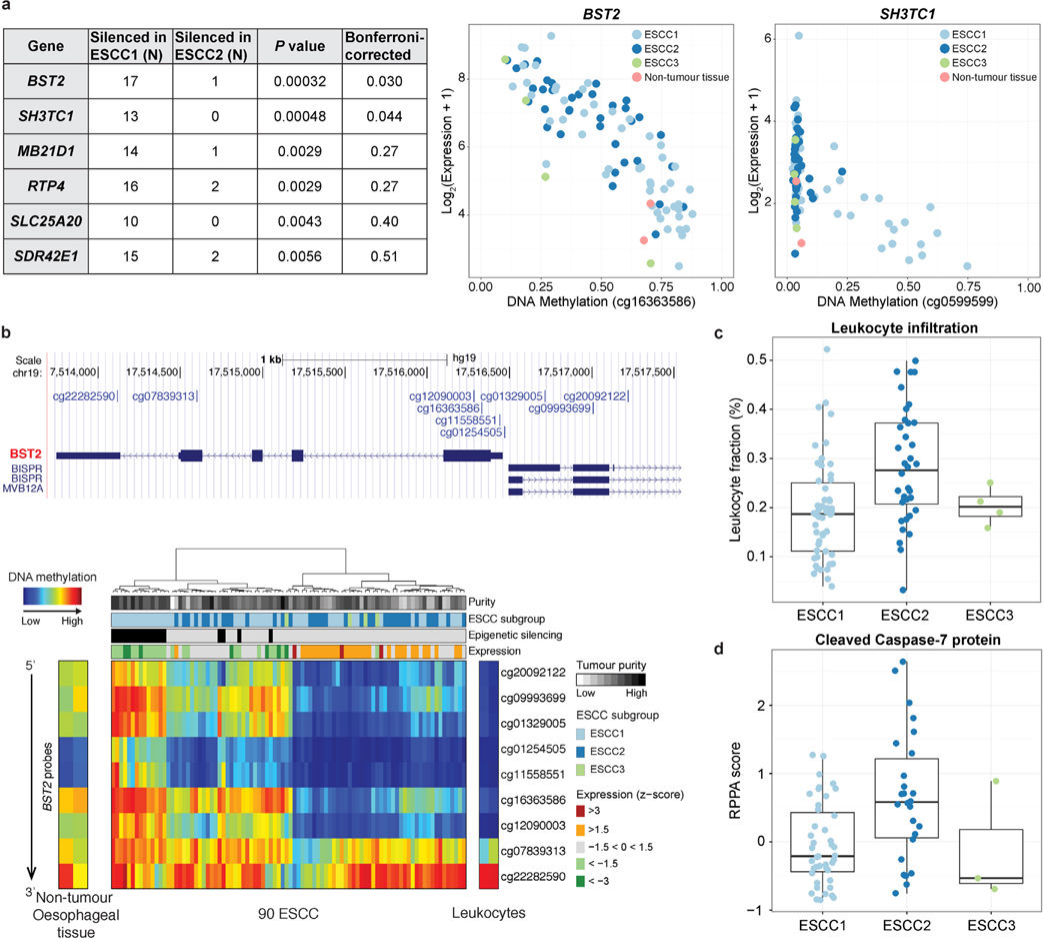
Characterization of ESCC subtypes **a**, We identified genes exhibiting epigenetic silencing in individual samples and compared the number of samples where each gene was silenced in ESCC1 and ESCC2. Genes that showed statistical associations between number of silenced samples and ESCC subtypes are shown in the table *(P* < 0.01, Fisher’s exact test). Two genes remained significant after Bonferroni correction. The panel on the right shows DNA methylation versus gene expression for *BST2* and *SH3TC1*. **b**, A detailed analysis of *BST2* DNA methylation in ESCC samples and non-cancer controls. **c**, **d**, The plots of (**c**) estimated leukocyte fraction and (**d**) levels of cleaved caspase-7 protein show the median, 25th and 75th percentile values (horizontal bar, bottom and top bounds of the box), and the highest and lowest values within 1.5 times the interquartile range (top and bottom whiskers, respectively).

ESCC2 showed higher rates of mutation of NOTCH1 or ZNF750 (Extended Data Fig. 5), more frequent inactivating alterations of KDM6A and KDM2D, CDK6 amplification, and inactivation of PTEN or PIK3R1. We found greater leukocyte infiltration of ESCC2 tumours and higher levels of cleaved Caspase-7 protein (Extended Data Fig. 7), the latter implying enhanced potential for XIAP-directed agents to facilitate apoptosis34. The gene with the lowest P value for the methylation difference between ESCC1 and ESCC2 was the immunomodulatory molecule BST2 (ref. 35) (P=3 × 10−4, Fisher’s exact test; Supplementary Table 4), which showed less methylation and higher expression in ESCC2 (Extended Data Fig. 7), suggesting potential for BST2 inhibition.

ESCC3 tumours showed no evidence for genetic deregulation of the cell cycle and had TP53 mutations in only one of four samples. All samples in ESCC3, however, sustained alterations predicted to activate the PI3K pathway (Extended Data Fig. 5), and three of four possessed somatic alterations of KMT2D/MLL2 in addition to SMARCA4. Analysis of the TCGA HNSCC data set revealed no tumours with profiles analogous to ESCC3, suggesting this class of squamous tumours may be confined to ESCC.

ESCC subtypes showed trends for geographic associations: tumours from Vietnamese patients, the only Asian population studied, tended to be ESCC1 (27 out of 41 = 66%; P = 0.09, Fisher’s exact test), and more tumours derived from Eastern European and South American patients were ESCC2 (P = 0.118, Fisher’s exact test). All four ESCC3 tumours were derived from patients from the USA and Canada (P = 0.001, Fisher’s exact test). Tumours from Vietnamese patients were enriched in NFE2L2 mutations (Fig. 3c); 24% in the Vietnamese cohort (10 out of 41) versus 6% in other patients (3 out of 49; P = 0.017, Fisher’s exact test). This association of NFE2L2 mutations with Vietnamese patients suggests a common oxidative stressor or genetic predisposition. Patients from East Asia have common variants in alcohol-metabolism genes ALDH2 and ADH1B36, which are associated with ESCC risk36, but we could not investigate their association with NFE2L2 mutations as all Vietnamese patients had such variants (Supplementary Fig. 3.3).

In comparison to EAC, ESCCs showed enrichment of C> A substitutions and APOBEC (apolipoprotein B mRNA editing enzyme, catalytic polypeptide-like) signatures (P = 7 × 10^−7^ and 5 × 10^−5^, respectively, by Wilcoxon rank-sum test). The C>A mutational signature is associated with smoking and chewing tobacco^37^, but did not correlate with ESCC subgroups or clinical variables in our sample set. However, when we restricted the analysis to lifelong nonsmokers, the C>A signature was significantly higher in our Vietnamese population (P = 0.013, Wilcoxon), suggesting a role for tobacco chewing. The APOBEC signature was overrepresented in ESCC2 (Fig. 3d, P = 0.03, Kruskal–Wallis test) and enriched in patients from Ukraine and Russia (P = 0.01, Wilcoxon rank-sum test). ESCC tumours lacked the predilection for A>C transversions at AA dinucleotides seen in EAC (Supplementary Table 5).

We evaluated whether the human papilloma virus (HPV), which has a pathogenic role in cervical SCC and HNSCC, also contributes to ESCC, as has been reported^38^. Comparison of ESCC mRNA sequencing data to TCGA HNSCC data found that ESCC HPV transcript levels resembled HPV-negative HNSCC tumours (Fig. 3e). These data do not support an aetiologic role for HPV in ESCC.

## EAC in relation to gastric cancer

**Extended Data Figure 8:**
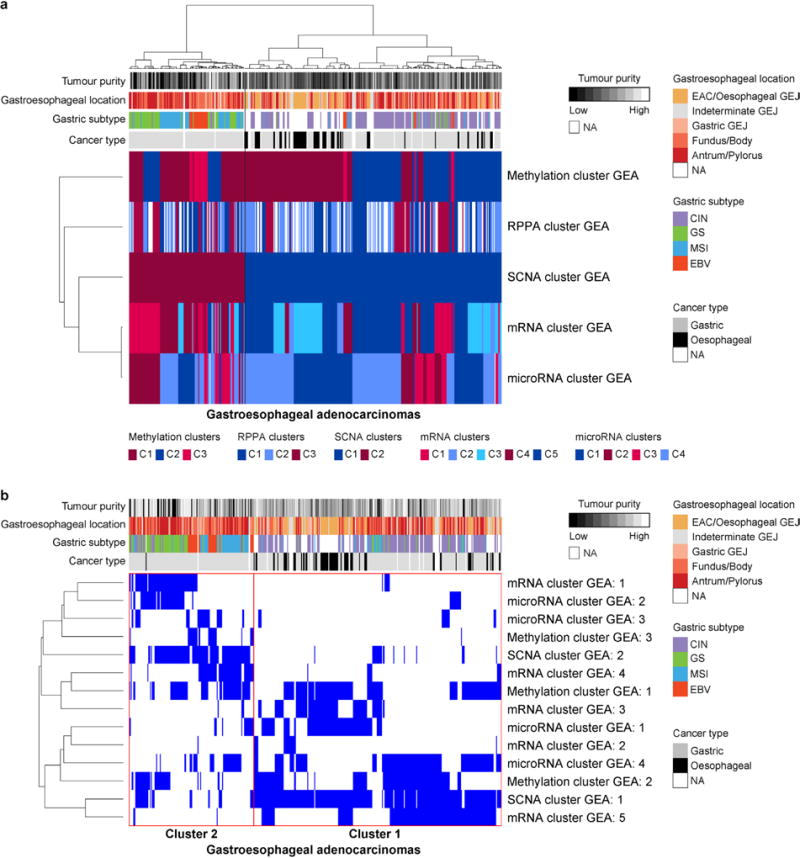
EACs are more similar to CIN-type gastric adenocarcinomas than to other gastric subtypes **a**, **b**, Integrative clustering of platform-specific clusters for gastroesophageal adenocarcinomas (GEA) was performed using the SuperCluster method (**a**) and Clustering of Cluster Assignments (COCA) (**b**).

**Figure 4:**
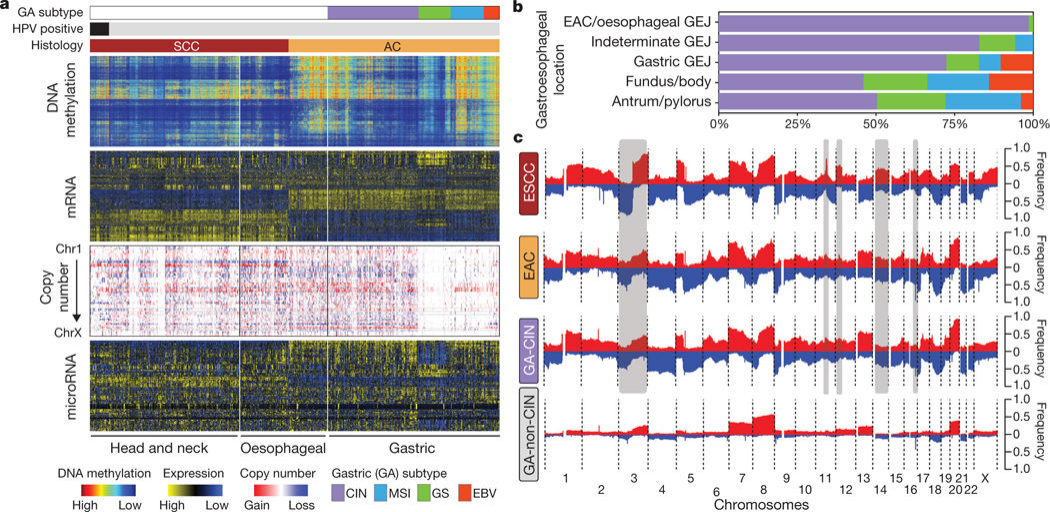
Similarity of oesophageal adenocarcinoma and CIN variant of gastric cancer **a**, Molecular profiles of head and neck, oesophageal and gastric carcinomas with samples segregated by tumour type and gastric cancers subdivided by molecular subtypes. **b**, Distribution of gastric molecular subtypes by anatomic location across gastroesophageal adenocarcinomas. **c**, Composite copy number profiles for ESCC, EAC, gastric-CIN and gastric non-CIN tumours with gains in red and losses in blue and grey highlighting differences between ESCC and EAC.

Given the uncertainty regarding appropriate demarcations of EAC relative to both gastric cancer and ESCC, we analysed both EAC and ESCC relative to the cancer types that occur nearest to the oesophagus, HNSCC and gastric adenocarcinoma. Analysis of mRNA expression, DNA methylation and SCNA data demonstrated that ESCC had a stronger resemblance to HNSCC than to EAC (Fig. 4a). Similarly, EACs more closely resembled gastric cancer than they did ESCC. In our previous TCGA study^27^, we classified gastric tumours into four subtypes on the basis of having (1) Epstein-Barr virus (EBV) infection, (2) microsatellite instability (MSI), (3) chromosomal instability (CIN) and (4) genomic stability (GS), a group largely comprised of the diffuse histologic type. When we evaluated EACs jointly with gastric cancers, we observed that EACs and CIN gastric cancers jointly formed a group distinct from EBV, MSI or GS tumours (Extended Data Fig. 8). Evaluating all gastroesophageal adenocarcinomas (GEAs), we found increasing prevalence of CIN moving proximally with 71 of 72 EACs classified as CIN (Fig. 4b). No EACs were positive for MSI or EBV. However, among GEJ adenocarcinomas that were not clearly of oesophageal origin, we identified MSI-positive and EBV-positive tumours.

**Extended Data Figure 9:**
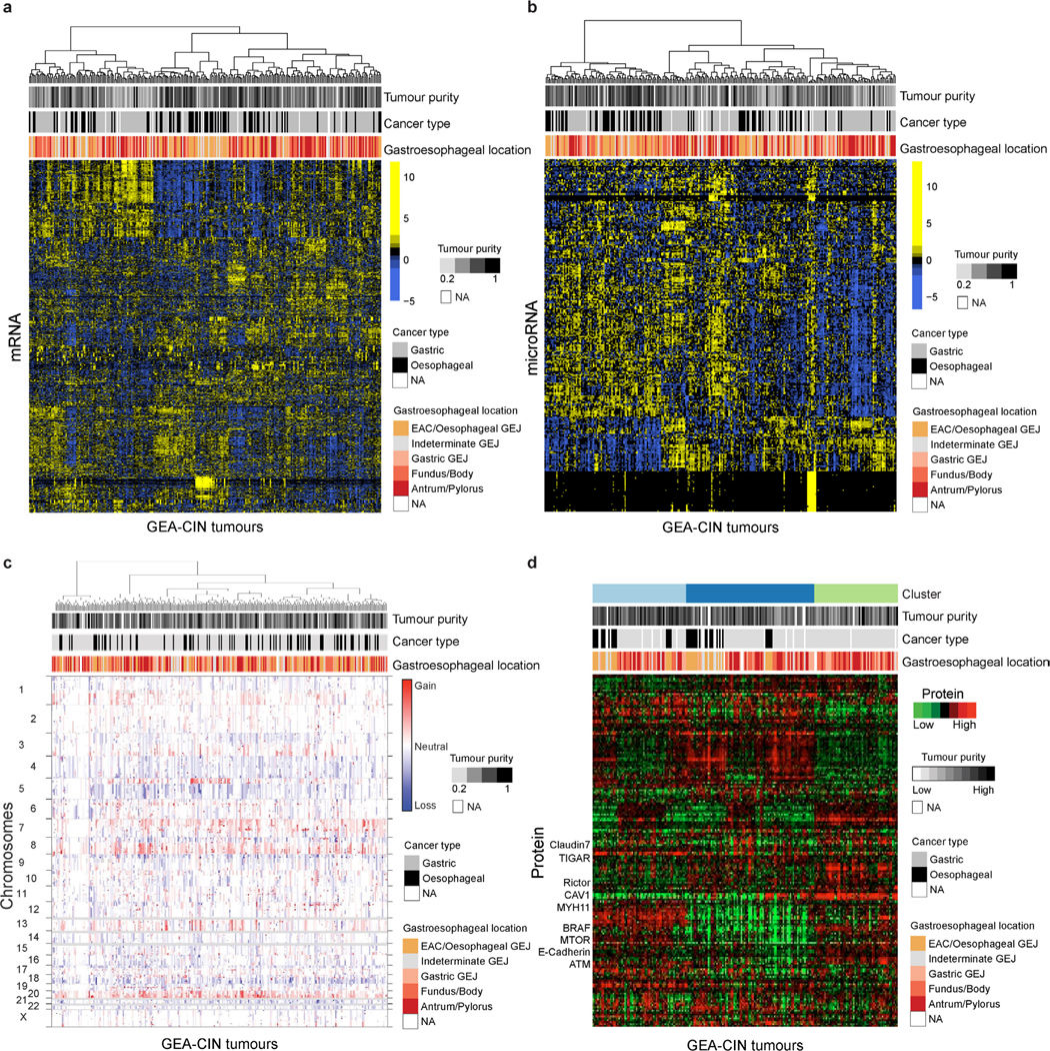
Platform-specific unsupervised clustering analyses of GEA-CIN tumours **a–d**, Shown are heat map representations of gene expression (**a**), microRNA (**b**), SCNAs (**c**), and reverse-phase protein array profiles of GEA-CIN tumours (columns) (**d**).

**Extended Data Figure 10:**
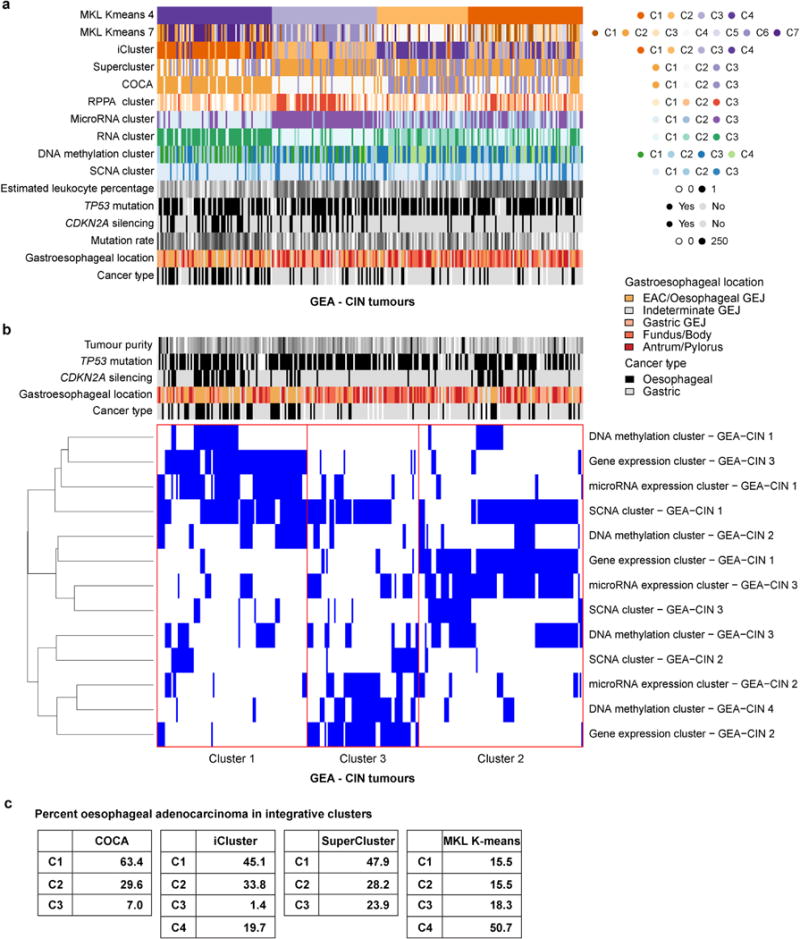
Integrative clustering of GEA-CIN samples **a**, Integrative clustering by Multiple Kernel Learning: *k*-means (MKL *k*-means) yielded a four cluster solution, in which Cluster 4 is enriched for EAC. **b**, Clustering of Cluster Assignments (COCA), was performed for the 267 samples for which complete platform-specific cluster information (see Fig. 5a, Extended Data Fig. 8) was available for gene expression, microRNA expression, DNA methylation and somatic copy number alteration (SCNA), and yielded three integrative clusters. Details of the methods can be found in Supplementary section S10.2. **c**, Frequency of EAC in four integrative clustering methods. Integrated clustering with iCluster and SuperCluster was performed as described in Methods.

The enrichment of CIN in EAC suggested that comparisons of EAC with gastric cancers would be confounded by non-CIN tumours nearly exclusively in the stomach. We therefore sought to find features that could differentiate EAC from CIN gastric cancers by analysis of the 288 CIN GEAs (GEA-CIN; Fig. 1a). We found clear similarity between chromosomal aberrations in gastric CIN tumours and EAC (Fig. 4c), with stronger similarity between EAC and CIN gastric cancers than between those of EAC and ESCC. Clustering of GEA-CIN data from individual platforms (Extended Data Fig. 9) and by integrative clustering revealed no consistent separation of EACs and CIN gastric cancers, thus arguing against classifying these as distinct diseases (Extended Data Fig. 10). As misannotation of tumours near the GEJ could enhance the apparent similarity of EACs and CIN gastric tumours, we repeated our analysis after excluding equivocal GEJ cases, but saw no definitive separation of EAC and CIN gastric adenocarcinomas (Supplementary Fig. 7.1).

**Figure 5:**
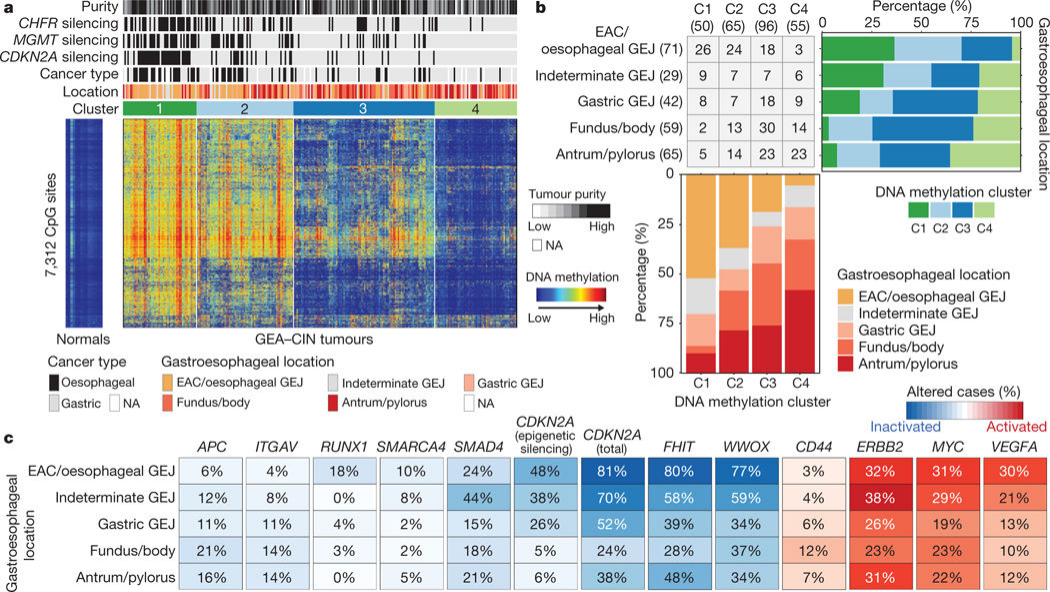
Molecular features of CIN gastroesophageal adenocarcinomas by anatomic location **a**, Heat map representation of consensus clustering of DNA methylation of GEA-CIN tumours with molecular and clinical features shown above and methylation profiles of normal oesophagus (*n* = 2) and stomach (*n* = 13) on the left. **b**, Fraction of tumours belonging to each methylation subgroup by anatomic location (top right) and distribution of tumour anatomic location by methylation cluster (bottom). **c**, Frequency of alterations in selected genes along the anatomic axis with tumour suppressor inactivation in blue and oncogene activation in red.

However, clustering of DNA methylation data revealed a progression of DNA methylation features from proximal to distal GEA-CIN tumours (Fig. 5a). Samples in cluster 1, those with the most frequent hypermethylation, were enriched in the oesophagus or proximal stomach/GEJ (Fig. 5b). The proportion of cancers showing more frequent DNA hypermethylation (that is, clusters 1 or 2) was significantly higher among EACs than among gastric CIN cancers (70% versus 30%, respectively; P = 1.0 × 10^−8^, Fisher’s exact test). By contrast, cluster 4, with the lowest rates of hypermethylation, included more distal stomach cancers (Fig. 5b). Unlike hypermethylated gastric CpG island methylator phenotype tumours, no GEA-CIN tumours exhibited epigenetic silencing of MLH1, consistent with their MSI-negative status, but they showed a higher propensity for epigenetic silencing of CDKN2A, (Supplementary Table 6, Fig. 5c). Additional genes silenced in cluster 1 included MGMT and CHFR, for which methylation has been associated with responses to alkylating agents and microtubule inhibitors, respectively^39,40^.

We evaluated the GEA-CIN tumours for somatic features that could differentiate EACs from gastric CIN tumours (Fig. 5c). EACs had higher rates of mutation of *SMARCA4* and deletion of tumour suppressor RUNX1, but lower APC mutation rates relative to gastric tumours, suggesting a less prominent role for Wnt/β-catenin in EAC. Copy-number analysis revealed higher rates of deletions of putative fragile site genes FHIT or WWOX, suggestive of differences in the underlying genomic instability between distal and proximal GEA-CIN tumours. Analysis of oncogenes identified subtle distinctions, with VEGFA and MYC amplifications being more common in EACs. Although additional samples will be required to refine understanding of the progressive gradations of features from the distal stomach to the oesophagus, these data indicate that gastric and oesophageal CIN tumours lack absolute dichotomizing features and do not appear to be distinct tumour types.

## Discussion

These analyses call into question the premise of envisioning oesophageal carcinoma as a single entity. These molecular data show that histological subtypes of EAC and ESCC are distinct in their molecular characteristics across all platforms tested. ESCC emerges as a disease more reminiscent of other SCCs than of EAC, which itself bears striking resemblance to CIN gastric cancer. Our analyses therefore argue against approaches that combine EAC and ESCC for clinical trials of neoadjuvant, adjuvant or systemic therapies (Supplementary Fig. 3.4).

**Figure 6:**
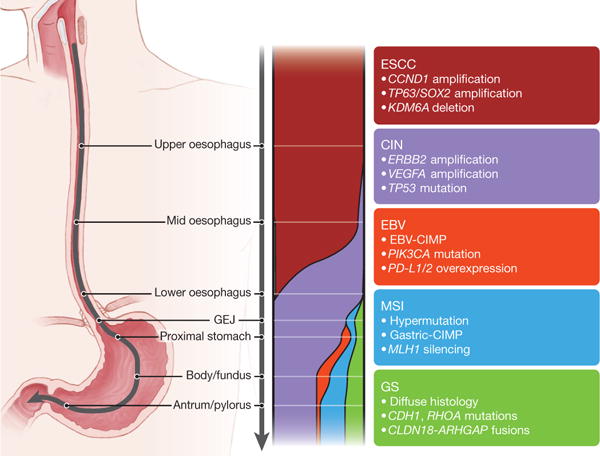
Gradations of molecular subclasses of gastroesophageal carcinoma Schematic representing shifting proportion of subtypes of gastroesophageal carcinoma from the proximal oesophagus to the distal stomach. The widths of the colour bands represent the proportion of the subtypes present within anatomic regions. Key features of subtypes are indicated in associated text.

These data also inform longstanding debates regarding appropriate demarcations of EAC from gastric cancer. We found that GEAs show a progressive gradation of subtypes (Fig. 6), with increasing prevalence of the CIN phenotype proximally, to the point that EACs appear to represent a disease of chromosomal instability. This CIN gradient is analogous to colorectal carcinomas, whereby CIN prevalence increases distally towards the rectum^41^. EAC has been considered separate from gastric cancer according to a model whereby EAC originates from Barrett’s oesophagus and thus is not of gastric origin. Although the origin of Barrett’s oesophagus remains controversial, recent mouse models suggest that Barrett’s oesophagus and EAC might originate from proximal gastric cells or embryonic remnant cell populations at the GEJ^42,43^. The notable molecular similarity between EACs and CIN gastric cancers provides indirect support for gastric origin of Barrett’s oesophagus and EAC and indicates that we may view GEA as a singular entity, analogously to colorectal adenocarcinoma. However, these similarities between EAC and CIN gastric cancers do not indicate that all CIN GEAs are indistinguishable. Indeed, differences in more proximal GEAs should be expected, given their distinct epidemiology, rapid increase in Western countries, and inverse association with *Helicobacter pylori*. Continued exploration of the molecular characteristics of EAC might not absolutely differentiate them from CIN gastric cancers, but may reveal additional features that are enriched in this variant of GEA.

## METHODS

### Data reporting

No statistical methods were used to predetermine sample size. The experiments were not randomized and the investigators were not blinded to allocation during experiments and outcome assessment.

### Specimen collection and staging

Tissue source sites (TSS) are listed in Supplementary Information S1.1. Oesophageal tumours were collected and shipped to a central Biospecimen Core Resource (BCR) between 1 December 2011 and 23 December 2013. Samples were obtained from patients who had received no previous chemotherapy or radiotherapy for their disease. Each frozen primary tumour specimen had a companion normal tissue specimen (blood or blood components, including DNA extracted at the TSS). Adjacent nontumourous oesophageal tissue was also submitted for a subset of patients.

Cases were staged according to the American Joint Committee on Cancer 7th edition staging system^44^. Pathology quality control was performed on each tumour and adjacent normal tissue specimen (if available) from a frozen section slide to confirm that the tumour specimen was histologically consistent with oesophageal cancer and that the adjacent tissue specimen contained no tumour cells. Tumour samples with ≥ 60% tumour nuclei and ≤ 20% necrosis were submitted for nucleic acid extraction.

### Nucleic acid processing and qualification

DNA and RNA were co-isolated, and quality was assessed at the central BCR as described previously (supplementary S1.1 in ref. 27). A custom Sequenom SNP panel or the AmpFISTR Identifiler (Applied Biosystems) was used to verify that tumour DNA and germline DNA representing a case were derived from the same patient. RNA was analysed through the RNA6000 Nano assay (Agilent) to determine an RNA Integrity Number, and only analytes with an integrity number ≥ 7.0 were included. Only cases yielding a minimum of 6.9 μg of tumour DNA, 5.15 μg of RNA and 4.9 μg of germline DNA were included.

The BCR received tumour samples with germline controls from a total of 322 oesophageal cancer cases, of which 185 qualified, on the basis of BCR pathology review and molecular characteristics. Distribution and quality control of cases is shown in Supplementary Fig. 1.1. Of the 185 cases that qualified, 171 cases were used for genomic analysis, as 14 cases were excluded after independent pathology review (described in ‘Expert pathology review’, below) or discovery of clinical or molecular disqualifiers.

Of the 171 qualifying cases, matched nontumourous oesophageal tissue was available for 58 cases. Samples with residual tumour tissue after extraction of nucleic acids were considered for proteomics analysis. When available, a 10- to 20-mg piece of snap-frozen tumour adjacent to the piece used for molecular sequencing and characterization was submitted for reverse-phase protein array analysis. We compared these 171 oesophageal adenocarcinomas to 388 similarly characterized gastric adenocarcinomas (Supplementary Fig. 1.1).

### Microsatellite instability assay

Microsatellite instability (MSI) in qualified oesophageal adenocarcinoma tumour-derived DNA samples was evaluated by the BCR at Nationwide Children’s Hospital, Columbus, Ohio, USA. MSI-mono-dinucleotide assay was performed to test a panel of four mononucleotide repeat loci (polyadenine tracts BAT25, BAT26, BAT40 and transforming growth factor receptor type II) and three dinucleotide repeat loci (CA repeats in D2S123, D5S346 and D17S250) as previously described^27^.

### Expert pathology review

All cancers included in this study were secondarily reviewed by an Expert Pathologists’ Committee that consisted of seven experienced gastrointestinal pathologists (R.O., S.McC., Z.Z., J.K., L.T., M.B.P. and J.W.). A centralized virtual pathology review system was constructed using an Aperio slide scanner housed at the BCR at Nationwide Children’s Hospital. Typically, two frozen sections flanking the tumour tissue from which all material was extracted for this study and one additional high-quality formalin-fixed paraffin-embedded tissue section were scanned and reviewed. Two committee members reviewed all cases before inclusion into the study. For cases with discrepant results, a tiebreaker reviewer was assigned.

All oesophageal cancers were categorized as squamous or adenocarcinoma, according to the World Health Organization Classification of Tumours of the Digestive System, 4th edition^45^. Nine cases were excluded on the basis of pathology review, including four cases where quality control identified inadequate material for analysis, two cases where only noninvasive neoplasm was observed, and two cases where the neoplasm was unclassifiable on the basis of the material available for review. As part of this review, an additional 77 gastric adenocarcinomas that had not undergone pathology review as part of this group’s original published analysis were also subject to pathology re-review as performed previously^27^.

Clinical staging was assessed^44^ by two reviewers according to criteria for each tumour type (ESCC or EAC). T, N and M status and tumour grade (0, 1, 2 or 3) were based on pathology reports from the TSS.

### Anatomic subclassification of adenocarcinomas involving the GEJ

All adenocarcinomas (oesophageal or gastric) from the TCGA collections that had a potential origin near the GEJ were further reviewed to refine their anatomic location. Pathology reports were obtained from the TSSs with the original gross pathology description of the tumour at resection or endoscopic biopsy. Two independent clinical reviewers reviewed each TSS pathology report. Tumours were classified as oesophageal, probable oesophageal, indeterminate, probable gastric or gastric, according to criteria outlined in Supplementary Information S1.2. For downstream analyses, the oesophageal and probable oesophageal were grouped together, as were the gastric and probable gastric.

### Somatic copy-number analysis

Analysis of SCNAs was performed on the basis of DNA profiling of each tumour or germline sample on Affymetrix SNP 6.0 at the Genome Analysis Platform of the Broad Institute as previously described^46^. As part of this process of copy-number assessment and segmentation, regions corresponding to germline copy-number alterations were removed by applying filters generated from either the TCGA germline samples from our ovarian cancer analysis or from samples in this collection. Analysis of recurrent broad and focal SCNAs was performed with the GISTIC 2.0 algorithm^22^ with clustering performed in R, on the basis of Euclidean distance using thresholded copy number at recurring alteration peaks from GISTIC analysis using Ward’s method, both as previously reported^27^. Allelic copy number and purity and ploidy estimates were calculated using the ABSOLUTE algorithm^47^. Tumours were classified as having high chromosomal instability, SCNA-high, if they possessed at least one arm-level loss (apart from that of 18p, 18q or 21, which were recurrent in tumours of both low and high copy-number events) and otherwise as SCNA-low. Chromosomal arms were considered altered if at least 80% of the arm was lost or gained with a relative log_2_ copy ratio change of at least 0.15 (Shih *et al.*, unpublished observations). This method of classifying copy number instability has 93% concordance with previously described copy-number clustering^27^.

### DNA methylation

Genomic DNA (1 μg per sample) was bisulfite-modified, subjected to quality control, and analysed using the Illumina Infinium DNA methylation platform, HumanMethylation450, as detailed in Supplementary Information S2. Data files generated are listed in Supplementary Information S2.3.

### *CDKN2A* epigenetic silencing calls

*CDKN2A* (also known as *p16INK4)* epigenetic silencing calls were made using both DNA methylation and RNA-seq data. *CDKN2A* DNA methylation status was assessed in each sample based on the probe (cg13601799) located in the p16INK4 promoter CpG island. p16INK4 expression was determined by the log_2_(RPKM+1) level of its first exon (chr9: 21974403–21975132). The epigenetic silencing calls for each sample were made by evaluating a scatterplot showing an inverse association between DNA methylation and expression as described in Supplementary Information S2.

### DNA sequence analysis

Exome and full-coverage whole-genome sequencing was split between two sequencing centres. Samples that were submitted to TCGA as stomach adenocarcinomas (that is, STAD, as labelled by the TSS) were sent for sequencing at the Broad Institute. Samples labelled as oesophageal cancers (that is, ESCA) were sequenced at Washington University. Each centre was responsible for generating BAM files from both tumour and normal DNA samples with additional filtering to remove likely artefacts of the sequencing process. From these BAM files, four different TCGA analysis sites performed distinct mutation and insertion/deletion detection procedures. The results of these distinct mutation-calling efforts were integrated to generate a common mutation annotation file for subsequent analysis. See Supplementary Section S3.1.

### Broad Institute sequencing

Whole-exome sequencing of 0.5 to 3 μg of DNA from tumour and normal blood samples was performed as previously described^32^ using the Agilent SureSelect Human All Exon V5 kit, followed by 2 × 76-bp paired-end sequencing on the Illumina HiSeq platform. For whole-genome sequencing, 2 × 101-bp reads were sequenced on the same platform. Read alignment and processing were performed using the Burrows–Wheeler Aligner (BWA) and Picard at the Broad Institute (http://broadinstitute.github.io/picard/) as previously published^27^. Alignments were first subjected to quality control using ContEst^48^ to avoid misannotation of tumour and germline DNA samples, or cross-contamination between tumour samples. Only samples with less than 5% estimated cross-contamination were analysed further.

### Washington University sequencing

Whole-exome sequencing and whole-genome Illumina libraries were constructed as described previously^49^ using Nimblegen SeqCap EZ Human Exome Library v3.0 combined with additional 120-mer IDT custom probes, targeting DNA from cancer-related viruses (for example, HPV, EBV) and sequenced in multiple lanes of Illumina HiSeq 2000 flow cells to achieve a minimum coverage of 20× across 80% of coding target exons. Each lane or sub-lane of data was aligned using BWA v0.5.9. to GRCh37-lite + accessioned target viruses(ftp://genome.wustl.edu/pub/reference/GRCh37-lite_WUGSC_variant_2/).

### Identification of somatic mutations and insertion/deletions

The BAM files (for exome sequencing) were used for mutation calling at four different analysis centres: Broad Institute, Washington University, University of California at Santa Cruz and British Columbia Cancer Agency (as detailed in Supplementary Methods S3.1).

Filtered calls from each analysis centre as described above were merged, and germline SNP sites reported by the 1000 Genomes project were filtered and removed. In addition, for the normal germline BAM, putative variants with less than 8× coverage of the reference allele or greater than one somatic variant-supporting read or 1% somatic variant allele fraction were removed. For the tumour BAM, two supporting reads and a variant allele fraction of 5% were required as a minimum. Filtering of putatively spurious mutation calls due to 8-oxoguanine artefacts was performed to remove candidate mutations attributed to these sequencing artefacts. Further filtering removed candidate mutations that had been identified through sequencing of cohorts of non-neoplastic DNA samples to remove alternative artefacts or unfiltered germline calls. Read counts were generated for all remaining novel putative variants, and these variants were incorporated into the final mutation annotation file if they met the same minimum coverage, maximum coverage, and variant allele fraction requirements described above.

### Mutation annotation and significance analysis

Functional annotation of mutations was performed with Oncotator (http://www.broadinstitute.org/cancer/cga/oncotator) using Gencode V18. Significantly recurrently mutated genes were identified using the MutSigCV2.0 algorithm^13^.

### Mutation signature analysis

Mutation signature discovery was performed using Bayesian non-negative matrix factorization algorithm for mutation signature analysis as described in Supplementary Information S3.2.

### Low-pass whole-genome sequencing for rearrangement identification

Genomic DNA (500–700 ng per sample) was sheared into 250-bp fragments using a Covaris E220 ultrasonicator, then converted to a paired-end Illumina library using KAPA Bio kits with Caliper (PerkinElmer) robotic NGS Suite (Partek Genomics) according to manufacturers’ protocols. All libraries were sequenced on a HiSeq2000 using one sample per lane, with a paired-end 2 × 51-bp read length. Tumour DNA and its matching normal DNA were usually loaded on the same flow cell. Raw data were converted to the FASTQ format, and BWA alignment (to hg19) was used to generate BAM files as previously described (supplementary S3.6 in ref. 27). Detection of structural rearrangements was performed using two algorithms, BreakDancer^50^ and Meerkat^51^. The set of structural variant calls from each tumour sample was filtered by the calls from its matched normal DNA to remove germline variants. Data were then re-examined using the Meerkat algorithm, which necessitated the identification of at least two discordant read pairs, with one read covering the actual breakpoint junction. Alterations found in simple or satellite repeats were also excluded. (Candidate fusion genes from this analysis are shown in Supplementary Table 3 with more detailed listing of structural alterations in Supplementary Table 7.)

### mRNA sequencing and analysis methods

mRNA sequence data were generated as described previously (supplementary S5.1 in ref. 27). For combined clustering analysis of oesophageal, gastric and head and neck tumours, the University of North Carolina Genome Characterization Center reprocessed the stomach adenocarcinoma and oesophageal cancer data with their MapSplice/RSEM pipeline^32^. We generated candidate fusion events from mRNA sequence data as described previously (supplementary S5.4 in ref. 27), except that we used TransABySS v1.4.8 (http://www.bcgsc.ca/platform/bioinfo/software/trans-abyss/releases/1.4.8).

To identify subtypes within our various cohorts, we used hierarchical clustering with pheatmap v1.0.2 in R. The input in each case was a reads per kilobase of exon per million reads mapped to the transcriptome (RPKM) data matrix for the top 25% most variable genes with mean greater than 10 RPKM. We transformed each row of the matrix by log_10_(RPKM+1), then used pheatmap to scale the rows. We used ward.D2 for the clustering method and correlation and Euclidean distance measures for clustering the columns and rows, respectively. We identified genes that were differentially expressed, using unpaired two-class significance analysis of microarrays (samr v2.0), with an RPKM input matrix and a false discovery rate threshold of 0.05.

To compare oesophageal cancer subtypes with established subtypes of HNSCC^52^ and lung squamous cell (LUSC) tumours^53^, centroid gene expression profiles were used to categorize the 90 oesophageal squamous tumours into atypical, basal, classical and mesenchymal by the HNSCC classification; and basal, classical, primitive and secretory by the LUSC classification. Of the 839 genes used for the HNSCC centroids, 809 overlapped with genes in the ESCC data set. Additionally, of the 209 genes used for the LUSC predictor centroids, 202 overlapped with genes in the ESCC data set. We then generated an RPKM matrix of the 90 ESCC tumour samples for each of these gene sets. These matrices were log_2_ transformed and median-centred. Finally, we computed the Pearson correlations between each column in the matrix and the HNSCC and LUSC centroids.

To evaluate oesophageal mRNA expression relative to other tumour types, we combined RNA sequencing by expectation maximization RSEM-normalized expression data from the STAD, ESCA and HNSC cohorts. Samples were ordered first by organ, then by histology (adenocarcinoma or squamous), then by gastric cancer classification (EBV, MSI, GS or CIN categories) and finally by HPV status. We selected the top 25% most variable genes (by coefficient of variation) within the oesophageal carcinoma sample set with mean expression greater than 1,000 RSEM-normalized counts. We transformed each row of the matrix by log_10_(RSEM+1), then used pheatmap to scale and cluster the rows.

### microRNA sequencing and analysis

We generated microRNA sequence data as described previously (supplementary S6.1 in ref. 27). To identify subtypes within our various cohorts, we used hierarchical clustering with pheatmap v1.0.2 in R. The input in each case was a reads-per-million (RPM) data matrix for the 303 miRBase v16 5p or 3p mature strands that had the largest variances across each cohort. We transformed each row of the matrix by log_10_(RPM+1), then used pheatmap to scale the rows. We used ward.D2 for the clustering method and correlation and Euclidean distance measures for clustering the columns and rows, respectively. For analyses comparing oesophageal with gastric and head and neck cancers, we used the top 25% (~300) most variable 5p or 3p mature strand microRNAs^54^ within the oesophageal carcinoma sample set. We transformed each row of the matrix by log_10_(RPM+1), then used pheatmap to scale the rows. For clustering the rows, we used ward.D2 and a Euclidean distance measure.

### Reverse-phase protein array

Proteins isolated from tumours were used to prepare reverse-phase protein arrays with 187 validated primary antibodies by methods described previously (supplementary S7 in ref. 27). Data were normalized, and clustering analysis was performed as detailed in Supplementary Section S4.

### Pathogen analysis

We used two tools to examine whole-exome and RNA sequence data for the presence of microbial sequences: BBT (BioBloomTools, v1.2.4.b1) and PathSeq. Details of these analyses are provided in Supplementary section S5. MicroRNA data were analysed using an in-house pipeline as previously described (supplementary S9.2 in ref. 27).

### Pathway analysis of mRNA

We performed pathway-level analysis of gene expression to compare EAC and ESCC samples. Pathways, as gene-sets, were obtained from the National Cancer Institute’s pathway interaction database (NCI-PID)^55^. A *P* value, comparing EAC with ESCC using Kruskal-Wallis one-way analysis of variance by ranks, was obtained for each gene. For each of the 224 pathways, the gene-level *p* values were log-transformed and summed by using an approach based on Fisher’s combined statistic to yield a pathway-level composite score. The statistical significance of this score was then estimated empirically by similarly scoring 10,000 randomly generated pathways for each NCI-PID pathway, with matched pathway size.

### Integrative clustering

To discover which tumour samples shared molecular signatures across platforms, the following four integrative clustering approaches were used: iCluster, Multiple Kernel Learning *k*-means (MKL *k*-means), SuperCluster, and Clustering of Cluster Assignments (COCA). In the iCluster method^10,56,57^, subgroups were discovered through their representation as latent variables in joint multivariate regression. MKL *k*-means combines the *k*-means clustering algorithm with the use of kernels that encode the similarity between the samples, to define features for classifying the tumours. SuperCluster and COCA both use clusters derived from individual molecular platforms to form an overall categorical description of each sample, but they differ in details, such as the metric used to compare those samples. SuperCluster performs a variance adjustment such that each molecular platform receives equal weight, whereas the implementation of COCA employed here and previously (supplementary S10.2 in ref. 27) uses a weighting method that takes into account the granularity of the divisions within each platform-specific category. Further details on these methods are given in Supplementary Section S7.

### Data availability

The primary and processed data used to generate the analyses presented here can be downloaded from the TCGA manuscript publication page, (https://tcga-data.nci.nih.gov/docs/publications/esca_2016), and from the Genomic Data Commons (https://gdc-portal.nci.nih.gov/legacy-archive).

## Extended Data

## Supplementary Material

Supp Table 1

Supp Table 2

Supp Table 3

Supp Table 4

Supp Table 5

Supp Table 6

Supp Table 7

Supplemental Data

## The Cancer Genome Atlas Research Network

**Analysis Working Group: Asan University** Jihun Kim^1^; **BC Cancer Agency** Reanne Bowlby^2^, Andrew J. Mungall^2^, A. Gordon Robertson^2^; Brigham and Women’s Hospital Robert D. Odze^3,4^; **Broad Institute** Andrew D. Cherniack^5^, Juliann Shih^5,6^, Chandra Sekhar Pedamallu^5,6^, Carrie Cibulskis^5^, Andrew Dunford^5^, Samuel R. Meier^5^, Jaegil Kim^5^; **Brown University** Benjamin J. Raphael^7^, Hsin-Ta Wu^7^, Alexandra M. Wong^7^; **Case Western Reserve University** Joseph E. Willis^89^; **Dana-Farber Cancer Institute** Adam J. Bass^6,10^, Sarah Derks^6,11^; Duke University Katherine Garman^12^, Shannon J. McCall^12^; Greater Poland Cancer Centre Maciej Wiznerowicz^13–15^; **Harvard Medical School** Angeliki Pantazi^16,17^, Michael Parfenov^16^; **Institute for Systems Biology** Vésteinn Thorsson^18^, Ilya Shmulevich^18^, Varsha Dhankani^18^, Michael Miller^18^; KU Leuven Ryo Sakai^19,20^; Mayo Clinic Kenneth Wang^21^; **Memorial Sloan Kettering Cancer Center** Nikolaus Schultz^22,23^, Ronglai Shen^23^, Arshi Arora^23^, Nils Weinhold^24^, Francisco Sánchez-Vega^22^, David P. Kelsen^25^; **National Cancer Institute** Julia Zhang^26^, Ina Felau^26^, John Demchok^26^, Charles S. Rabkin^27^, M. Constanza Camargo^27^, Jean Claude Zenklusen^26^; **Nationwide Children’s Hospital** Jay Bowen^28^, Kristen Leraas^28^, Tara M. Lichtenberg^28^; Stanford University Christina Curtis^29^, Jose A. Seoane^29^; **University of Alabama** Akinyemi I. Ojesina^30,31^; **University of Michigan** David G. Beer^32^; **University of North Carolina** Margaret L. Gulley^33^; **University of Pittsburgh** Arjun Pennathur^34^, James D. Luketich^34^; **University of Rochester** Zhongren Zhou^35^; **University of Southern California** Daniel J. Weisenberger^36^; **University of Texas MD Anderson Cancer Center** Rehan Akbani^37^, Ju-Seog Lee^38^, Wenbin Liu^37^, Gordon B. Mills^38^, Wei Zhang^39^; **University of Washington** Brian J Reid^40^; **Van Andel Research Institute** Toshinori Hinoue^41^, Peter W. Laird^41^, Hui Shen^41^; **Vanderbilt University** M. Blanca Piazuelo^42^, Barbara G. Schneider^42^; **Washington University** Michael McLellan^43^, **Genome Sequencing Center: Broad Institute** Amaro Taylor-Weiner^5,44^, Carrie Cibulskis^5^, Michael Lawrence^5^, Kristian Cibulskis^5^, Chip Stewart^5^, Gad Getz^5,45^, Eric Lander^5^, Stacey B. Gabriel^5^; **Washington University in St. Louis** Li Ding^43,46^, Michael D. McLellan^43^, Christopher A. Miller^43^, Elizabeth L. Appelbaum^43^, Matthew G. Cordes^43^, Catrina C. Fronick^43^, Lucinda A. Fulton^43^, Elaine R. Mardis^43^, Richard K. Wilson^43^, Heather K. Schmidt^43^, Robert S. Fulton^43^, **Genome Characterization Centers: BC Cancer Agency** Adrian Ally^2^, Miruna Balasundaram^2^, Reanne Bowlby^2^, Rebecca Carlsen^2^, Eric Chuah^2^, Noreen Dhalla^2^, Robert A. Holt^2^, Steven J. M. Jones^2^, Katayoon Kasaian^2^, Denise Brooks^2^, Haiyan I. Li^2^, Yussanne Ma^2^, Marco A. Marra^2^, Michael Mayo^2^, Richard A. Moore^2^, Andrew J. Mungall^2^, Karen L. Mungall^2^, A. Gordon Robertson^2^, Jacqueline E. Schein^2^, Payal Sipahimalani^2^, Angela Tam^2^, Nina Thiessen^2^, Tina Wong^2^; **Broad Institute** Andrew D. Cherniack^5,6^, Juliann Shih^5,6^, Chandra Sekhar Pedamallu^5^, Rameen Beroukhim^5,6,47^, Susan Bullman^5,6^, Carrie Cibulskis^5^, Bradley A. Murray^5^, Gordon Saksena^5^, Steven E. Schumacher^5,48^, Stacey Gabriel^5^, Matthew Meyerson^4,6^; **Harvard Medical School** Angela Hadjipanayis^16,49^, Raju Kucherlapati^16,49^, Angeliki Pantazi^16,17^, Michael Parfenov^16^, Xiaojia Ren^16,17^, Peter J. Park^44,49^, Semin Lee^44^, Melanie Kucherlapati^16,49^, Lixing Yang^44^; **The Sidney Kimmel Comprehensive Cancer Center at Johns Hopkins University** Stephen B. Baylin^50^; **University of North Carolina** Katherine A. Hoadley^51^; **University of Southern California Epigenome Center** Daniel J. Weisenberger^36^, Moiz S. Bootwalla^52^, Phillip H. Lai^52^, David J. Van Den Berg^53^, Mario Berrios^52^, Andrea Holbrook^52^; **University of Texas MD Anderson Cancer Center** Rehan Akbani^37^, Jun-Eul Hwang^54,55^, Hee-Jin Jang^54^, Wenbin Liu^37^, John N. Weinstein^37^, Ju-Seog Lee^54^, Yiling Lu^38^, Bo Hwa Sohn^54^, Gordon Mills^38^, Sahil Seth^56^, Alexei Protopopov^17,56^, Christopher A. Bristow^56^, Harshad S. Mahadeshwar^56^, Jiabin Tang^56^, Xingzhi Song^56^, Jianhua Zhang^56^; **Van Andel Research Institute** Peter W. Laird^41^, Toshinori Hinoue^41^, Hui Shen^41^, **Genome Data Analysis Centers: Broad Institute** Juok Cho^5^, Timothy Defrietas^5^, Scott Frazer^5^, Nils Gehlenborg^5,44^, David I. Heiman^5^, Michael S. Lawrence^5^, Pei Lin^5^, Samuel R. Meier^5^, Michael S. Noble^5^, Doug Voet^5^, Hailei Zhang^5^, Jaegil Kim^5^, Paz Polak^5,45^, Gordon Saksena^5^, Lynda Chin^5,56^, Gad Getz^5,45^; **Brown University**: Alexandra M. Wong^7^, Benjamin J. Raphael^7^, Hsin-Ta Wu^7^; **Harvard Medical School** Semin Lee^44^, Peter J. Park^44,49^, Lixing Yang^44^; **Institute for Systems Biology** Vésteinn Thorsson^18^, Brady Bernard^18^, Lisa Iype^18^, Michael Miller^18^, Sheila M. Reynolds^18^, Ilya Shmulevich^18^, Varsha Dhankani^18^; **Memorial Sloan Kettering Cancer Center** Adam Abeshouse^24^, Arshi Arora^23^, Joshua Armenia^22^, Ritika Kundra^22^, Marc Ladanyi^57^, Kjong-Van Lehmann^24^, Jianjiong Gao^22^, Chris Sander^24^, Nikolaus Schultz^22,23^, Francisco Sánchez-Vega^22^, Ronglai Shen^23^, Nils Weinhold^24^, Debyani Chakravarty^22^, Hongxin Zhang^22^; **University of California Santa Cruz** Amie Radenbaugh^58^; **University of Texas MD Anderson Cancer Center** Apruva Hegde^37^, Rehan Akbani^37^, Wenbin Liu^37^, John N. Weinstein^37^, Lynda Chin^5,56^, Christopher A. Bristow^56^, Yiling Lu^38^; **Biospecimen Core Resource: International Genomics Consortium** Robert Penny^59^, Daniel Crain^59^, Johanna Gardner^59^, Erin Curley^59^, David Mallery^59^, Scott Morris^59^, Joseph Paulauskis^59^, Troy Shelton^59^, Candace Shelton^59^; **The Research Institute at Nationwide Children’s Hospital** Jay Bowen^28^, Jessica Frick^28^, Julie M. Gastier-Foster^28^, Mark Gerken^28^, Kristen M. Leraas^28^, Tara M. Lichtenberg^28^, Nilsa C. Ramirez^28^, Lisa Wise^28^, Erik Zmuda^28^, **Tissue Source Sites: Analytic Biologic Services** Katherine Tarvin^60^, Charles Saller^60^; **Asan Medical Center** Young Soo Park^1^; **Asterand Bioscience** Michael Button^61^; **Barretos Cancer Hospital** Andre L. Carvalho^62^, Rui Manuel Reis^62,63^; Marcus Medeiros Matsushita^64^, Fabiano Lucchesi^65^, Antonio Talvane de Oliveira^66^; **BioreclamationIVT** Xuan Le^67^; **Botkin Municipal Clinic** Oxana Paklina^68^, Galiya Setdikova^68^; **Chonnam National University Medical School** Jae-Hyuck Lee^69^; **Christiana Care Health System** Joseph Bennett^70^, Mary Iacocca^70^, Lori Huelsenbeck-Dill^70^; Cureline Olga Potapova^71^, Olga Voronina^71^, Ouida Liu^71^, Victoria Fulidou^71^; **Duke University** Crystal Cates^12^, Alexis Sharp^12^; **Emory University** Madhusmitara Behera^72^, Seth Force^72^, Fadio Khuri^72^, Taofeek Owonikoko^72^, Allan Pickens^72^, Suresh Ramalingam^72^, Gabriel Sica^72^; **Erasmus University Winand** Dinjens^73^, Anna van Nistelrooij^74,75^, Bas Wijnhoven^74^; **Indiana University School of Medicine** George Sandusky^76^; **Institute of Oncology of Moldova** Serghei Stepa^77^; **International Genomics Consortium** Daniel Crain^59^, Joseph Paulauskis^59^, Robert Penny^59^, Johanna Gardner^59^, David Mallery^59^, Scott Morris^59^, Troy Shelton^59^, Candace Shelton^59^, Erin Curley^59^; Invidumed Hartmut Juhl^78^; **Israelitisches Krankenhaus Hamburg** Carsten Zornig^79^; **Keimyung University School of Medicine** Sun Young Kwon^80^; **Memorial Sloan Kettering Cancer Center** David Kelsen^25^; **National Cancer Center** Goyang Hark Kyun Kim^81^; **Ontario Tumour Bank** John Bartlett^82^, Jeremy Parfitt^83^, Runjan Chetty^84^, Gail Darling^84^, Jennifer Knox^84^, Rebecca Wong^84^, Haila El-Zimaity^84^, Geoffrey Liu^84^; **Peter MacCallum Cancer Centre** Alex Boussioutas^85^; **Pusan National University Medical School** Do Young Park^86^; **Ribeirão Preto Medical School** Rafael Kemp^87^, Carlos Gilberto Carlotti Jr^87^, Daniela Pretti da Cunha Tirapelli^87^, Fabiano Pinto Saggioro^88^, Ajith Kumar Sankarankutty^87^, Houtan Noushmehr^89^, Jose Sebastião dos Santos^87^, Felipe Amstalden Trevisan^87^; **St. Joseph’s Hospital & Medical Center** Jennifer Eschbacher^90^; **St. Petersburg Academic University** Michael Dubina^91^, Eugene Mozgovoy^91^; **Tayside Tissue Bank** Frank Carey^92^, Sally Chalmers^92^; **University of Dundee** Ian Forgie^92^; **University of Kansas Medical Center** Andrew Godwin^93^, Colleen Reilly^93^, Rashna Madan^93^, Zaid Naima^93^; **University of Michigan** Daysha Ferrer-Torres^32^, Michele Vinco^94^; **University of North Carolina at Chapel Hill** W. Kimryn Rathmell^95^; **University of Pittsburgh School of Medicine** Rajiv Dhir^96^, James Luketich^34^, Arjun Pennathur^34^; **University of Texas MD Anderson Cancer Center** Jaffer A. Ajani^97^, **Disease Working Group: Duke University** Shannon J. McCall^12^; **Memorial Sloan Kettering Cancer Center** Yelena Janjigian^25^, David Kelsen^25^, Marc Ladanyi^57^, Laura Tang^57^; **National Cancer Institute** M. Constanza Camargo^27^; **University of Texas MD Anderson Cancer Center** Jaffer A. Ajani^97^; **Yonsei University College of Medicine** Jae-Ho Cheong^98^, **Data Coordination Center: CSRA Inc**. Sudha Chudamani^99^, Jai Liu^99^, Laxmi Lolla^99^, Rashi Naresh^100^, Todd Pihl^100^, Qiang Sun^100^, Yunhu Wan^100^, Ye Wu^99^, **Project Team: National Institutes of Health** John A. Demchok^26^, Ina Felau^26^, Martin L. Ferguson^26^, Kenna R. Mills Shaw^26,54^, Margi Sheth^26^, Roy Tarnuzzer^26^, Zhining Wang^26^, Liming Yang^26^, Jean Claude Zenklusen^26^, Carolyn M. Hutter^101^, Heidi J. Sofia^101^, Jiashan Zhang^26^

^1^Department of Pathology, University of Ulsan College of Medicine, Asan Medical Center, Songpa-gu, Seoul 05505, Korea. ^2^Canada’s Michael Smith Genome Sciences Centre, BC Cancer Agency, Vancouver, BC V5Z 4S6, Canada. ^3^Department of Pathology, Brigham and Women’s Hospital, Boston, Massachusetts 02115, USA. ^4^Department of Pathology, Harvard Medical School, Boston, Massachusetts 02215, USA. ^5^The Eli and Edythe L. Broad Institute of Massachusetts Institute Of Technology and Harvard University, Cambridge, Massachusetts 02142, USA. ^6^Department of Medical Oncology, Dana-Farber Cancer Institute, Boston, Massachusetts 02115, USA. ^7^Department of Computer Science & Center for Computational Molecular Biology, Brown University, Providence, Rhode Island 02912, USA. ^8^Department of Pathology, Case Western Reserve University, Cleveland, Ohio 44106, USA. ^9^Dept of Pathology, Case Medical Center, Cleveland, Ohio 44106, USA. ^10^Center for Cancer Genome Discovery, Dana-Farber Cancer Institute, Boston, Massachusetts 02115, USA. ^11^Department of Medical Oncology, VU University Medical Center, Amsterdam, The Netherlands. ^12^Department of Pathology, Duke University, Durham, North Carolina 27710, USA. ^13^International Institute for Molecular Oncology, 60-203 Poznań, Poland. ^14^Greater Poland Cancer Centre, Poznań, 61-866, Poland. ^15^Poznan University of Medical Sciences, 61-866 Poznań, Poland. ^16^Department of Genetics, Harvard Medical School, Boston, Massachusetts 02115, USA. ^17^KEW Group Inc., Cambridge, Massachusetts 02139, USA. ^18^Institute for Systems Biology, Seattle, Washington 98109, USA. ^19^KU Leuven, Department of Electrical Engineering-ESAT(STADIUS), Leuven, Belgium. ^20^iMinds Medical IT, KU Leuven 3001, Belgium. ^21^Division of Gastroenterology and Hepatology, Mayo Clinic, Rochester, Minnesota 55905, USA. ^22^Center for Molecular Oncology, Memorial Sloan Kettering Cancer Center, New York, New York 10065, USA. ^23^Department of Epidemiology and Biostatistics, Memorial Sloan Kettering Cancer Center, New York, New York 10065, USA. ^24^Computational Biology Center, Memorial Sloan Kettering Cancer Center, New York, New York 10065, USA. ^25^Department of Medicine, Memorial Sloan Kettering Cancer Center, New York, New York 10065, USA. ^26^National Cancer Institute, Bethesda, Maryland 20892, USA. ^27^Division of Cancer Epidemiology and Genetics, National Cancer Institute, Bethesda, Maryland 20892, USA. ^28^The Research Institute at Nationwide Children’s Hospital, Columbus, Ohio 43205, USA. ^29^Department of Medicine, Division of Oncology and Department of Genetics, Stanford University School of Medicine, Stanford, California 94305, USA. ^30^Department of Epidemiology, University of Alabama at Birmingham, Birmingham, Alabama 35294, USA. ^31^HudsonAlpha Institute for Biotechnology, Huntsville, Alabama 35806, USA. ^32^Department of Thoracic Surgery, University of Michigan Comprehensive Cancer Center, Ann Arbor, Michigan 48109, USA. ^33^Department of Pathology and Laboratory Medicine, University of North Carolina at Chapel Hill, Chapel Hill, North Carolina 27599, USA. ^34^Department of Cardiothoracic Surgery, University of Pittsburgh Medical Center, University of Pittsburgh School of Medicine, Pittsburgh, Pennsylvania 15213, USA. ^35^Department of Pathology and Laboratory Medicine, University of Rochester, Rochester, New York 14642, USA. ^36^Department of Biochemistry and Molecular Biology, University of Southern California, Los Angeles, California 90033 USA. ^37^Department of Bioinformatics and Computational Biology, The University of Texas MD Anderson Cancer Center, Houston, Texas 77030, USA. ^38^Department of Systems Biology, The University of Texas MD Anderson Cancer Center, Houston, Texas, 77030, USA. ^39^Department of Pathology, The University of Texas MD Anderson Cancer Center, Houston, Texas 77030, USA. ^40^Fred Hutchinson Cancer Research Center, North Seattle, Washington 98109, USA. ^41^Center for Epigenetics, Van Andel Research Institute, Grand Rapids, Michigan 49503, USA. ^42^Department of Medicine, Division of Gastroenterology, Vanderbilt University Medical Center, Nashville, Tennessee 37232, USA. ^43^McDonnell Genome Institute at Washington University, St. Louis, Missouri 63108, USA. ^44^Department of Biomedical Informatics, Harvard Medical School, Boston, Massachusetts 02115, USA. ^45^Department of Pathology, Massachusetts General Hospital, Boston, Massachusetts 02114, USA. ^46^Department of Medicine, Washington University School of Medicine, St. Louis, Missouri 63108, USA. ^47^Department of Medicine, Harvard Medical School, Boston, Massachusetts 02215, USA. ^48^Department of Cancer Biology, Dana-Farber Cancer Institute, Boston, Massachusetts 02215, USA. ^49^Division of Genetics, Brigham and Women’s Hospital, Boston, Massachusetts 02115, USA. ^50^Cancer Biology Division, Sidney Kimmel Comprehensive Cancer Center at Johns Hopkins University, Baltimore, Maryland 21231, USA. ^51^University of North Carolina, Lineberger Comprehensive Cancer Center, Chapel Hill, North Carolina 27514, USA. ^52^University of Southern California, USC/Norris Comprehensive Cancer Center, Los Angeles, California 90033, USA. ^53^Department of Preventive Medicine, University of Southern California, Los Angeles, California 90033, USA. ^54^Institute for Personalized Cancer Treatment, Department of Systems Biology, The University of Texas MD Anderson Cancer Center, Houston, Texas 77030, USA. ^55^Department of Hemato-Oncology, Chonnam National University Medical School, Kwangju, Korea. ^56^Institute for Applied Cancer Science, Department of Genomic Medicine, University of Texas MD Anderson Cancer Center, Houston, Texas 77054, USA. ^57^Department of Pathology, Memorial Sloan Kettering Cancer Center, New York, New York 10065, USA. ^58^University of California Santa Cruz Genomics Institute, Santa Cruz, California 95064, USA. ^59^International Genomics Consortium, Phoenix, Arizona 85004, USA. ^60^Analytical Biological Services, Inc., Wilmington, Delaware 19801, USA. ^61^Asterand Bioscience, Detroit, Michigan 48202, USA. ^62^Molecular Oncology Research Center, Barretos Cancer Hospital, Barretos, São Paulo, Brazil. ^63^Life and Health Sciences Research Institute (ICVS), School of Health Sciences, University of Minho, Braga, Portugal. ^64^Department of Pathology, Barretos Cancer Hospital, Barretos, São Paulo, Brazil. ^65^Department of Radiology, Barretos Cancer Hospital, Barretos, São Paulo, Brazil. ^66^Department of Surgery, Barretos Cancer Hospital, Barretos, São Paulo, Brazil. ^67^Department of Research Pathology, BioreclamationIVT, Chestertown, Maryland 21620, USA. ^68^Botkin Municipal Clinic, Moscow 125284, Russia. ^69^Department of Pathology, Chonnam National University Medical School, Hwasun, Republic of Korea. ^70^Helen F Graham Cancer Center & Research Institute, Christiana Care Health System, Newark, Delaware 19713, USA. ^71^Cureline Inc, South San Francisco, California 94080, USA. ^72^Emory University and Winship Cancer Institute, Atlanta, Georgia 30322, USA. ^73^Department of Pathology, Erasmus MC Cancer Institute, University Medical Center, Rotterdam, 3000 CA Rotterdam, The Netherlands. ^74^Department of Surgery, Erasmus MC Cancer Institute, University Medical Center Rotterdam, 3000 CA Rotterdam, The Netherlands. ^75^Department of Pathology, Erasmus MC Cancer Institute, University Medical Center Rotterdam, 3000 CA Rotterdam, The Netherlands. ^76^Department of Pathology & Laboratory Medicine, Indiana University School of Medicine, Indianapolis, Indiana 46202, USA. ^77^Institute of Oncology of Moldova, Chisinau, Moldova. ^78^Indivumed GmbH, 20251 Hamburg, Germany. ^79^Israelitisches Krankenhaus Hamburg, 22297 Hamburg, Germany. ^80^Department of Pathology, Keimyung University School of Medicine, Daegu, Republic of Korea. ^81^Center for Gastric Cancer, National Cancer Center, Goyang, Republic of Korea. ^82^Ontario Tumour Bank, Ontario Institute for Cancer Research, Toronto, Ontario M5G 0A3, Canada. ^83^Ontario Tumour Bank, London Health Science Centre, London N6A 5A5, Canada. ^84^Princess Margaret Cancer Centre, Toronto, Ontario M5G2M9, Canada. ^85^Sir Peter MacCallum Cancer Department of Oncology, University of Melbourne, Melbourne 3002, Australia. ^86^Department of Pathology, Pusan National University Medical School, Pusan, Republic of Korea. ^87^Department of Surgery and Anatomy, Ribeirão Preto Medical School-FMRP, University of São Paulo, 14049-900, Brazil. ^88^Department of Pathology, Ribeirão Preto Medical School-FMRP, University of São Paulo, 14049-900, Brazil. ^89^Department of Genetics, Ribeirão Preto Medical School-FMRP, University of São Paulo, 14049-900, Brazil. ^90^Department of Pathology, St. Joseph’s Hospital and Medical Center, Phoenix, Arizona 85013, USA. ^91^St. Petersburg Academic University RAS, St. Petersburg 194021, Russia. ^92^Tayside Tissue Bank, University of Dundee, Ninewells Hospital and Medical School, Dundee DD1 9SY, UK. ^93^University of Kansas Medical Center, Kansas City, Kansas 66160, USA. ^94^Department of Pathology, University of Michigan, Ann Arbor, Michigan 48109, USA. ^95^Department of Medicine, Lineberger Comprehensive Cancer Center, University of North Carolina at Chapel Hill, Chapel Hill, North Carolina 27599, USA. ^96^Department of Pathology, University of Pittsburgh, Pittsburgh, Pennsylvania 15213, USA. ^97^Department of GI Medical Oncology, University of Texas MD Anderson Cancer Center, Houston, Texas 77030, USA. ^98^Department of Surgery, Yonsei University College of Medicine, Seoul, 120-752, Korea. ^99^Leidos Biomedical, Rockville, Maryland 20850, USA. ^100^CSRA Inc., Falls Church, Virginia 22042, USA. ^101^National Human Genome Research Institute, National Institutes of Health, Bethesda, Maryland 20892, USA.
