# Supplementary material for: Integrated genomic characterization of oesophageal carcinoma
Source: Nature. Author manuscript; Available in PMC 2017 Oct 22. (PMC5651175; doi:10.1038/nature20805)
Supplement: Supplemental Data [file NIHMS905030-supplement-Supplemental_Data.pdf]

SUPPLEMENTARY INFORMATION

S.1 Biospecimen collection, clinical data and pathology review

S1.1. Biospecimen collection, quality control and processing.

Specimens for The Cancer Genome Atlas (TCGA) oesophageal carcinoma (ESCA) project were shipped overnight from 35 tissue source sites (TSSs) using a cryoport that maintained an average temperature of less than -180°C. TSSs contributing biospecimens included Analytical Biological Services, Inc. (Indianapolis, IN, USA); Asan Medical Center (Seoul, Korea); Asterand Biosciences, Inc. (Detroit, MI, USA); BioreclamationIVT (Chestertown, MD, USA); Barretos Cancer Hospital (Barretos, Brazil); Botkin Municipal Clinic (Moscow, Russia); Chonnam National University Medical School (Hwasun, Korea); Christiana Care Health Services, Inc. (Newark, DE, USA); Cureline, Inc. (South San Francisco, CA, USA); Duke University (Durham, NC, USA); Emory University (Atlanta, GA, USA); Erasmus Medical Center (Rotterdam, Netherlands); ILSbio, LLC. (Chestertown, MD, USA); Indiana University School of Medicine (Indianapolis, IN, USA); Institute of Oncology of Moldova (Chisinau, Moldova); International Genomics Consortium (Phoenix, AZ, USA); Invidumed (Hamburg, Germany); Israelitisches Krankenhaus Hamburg (Hamburg, Germany); Keimyung University School of Medicine (Daegu, Korea); MD Anderson (Houston, TX, USA); Memorial Sloan Kettering Cancer Center (New York, NY, USA); National Cancer Center (Goyang, Korea); Ontario Institute for Cancer Research (Ottawa, ON, Canada); Peter MacCallum Cancer Center (Melbourne, Victoria, Australia); Pusan National University Medical School, (Pusan, Korea); Ribeirão Preto Medical School (São Paulo, Brazil); St. Joseph's Hospital and Medical Center (Phoenix, AZ, USA); St. Petersburg Academic University (St. Petersburg, Russia); Tayside Tissue Bank (Dundee, Scotland); University Health Network (Toronto, ON, Canada); University of Kansas Medical Center (Kansas City, KS, USA); University of Michigan (Ann Arbor, MI, USA); University of North Carolina at Chapel Hill (Chapel Hill, NC, USA); University of Pittsburgh (Pittsburgh, PA, USA); and University of São Paulo (São Paulo, Brazil). Analyses in this study were complemented with the use of cases from TCGA study of stomach cancer[1], referred to as STAD.

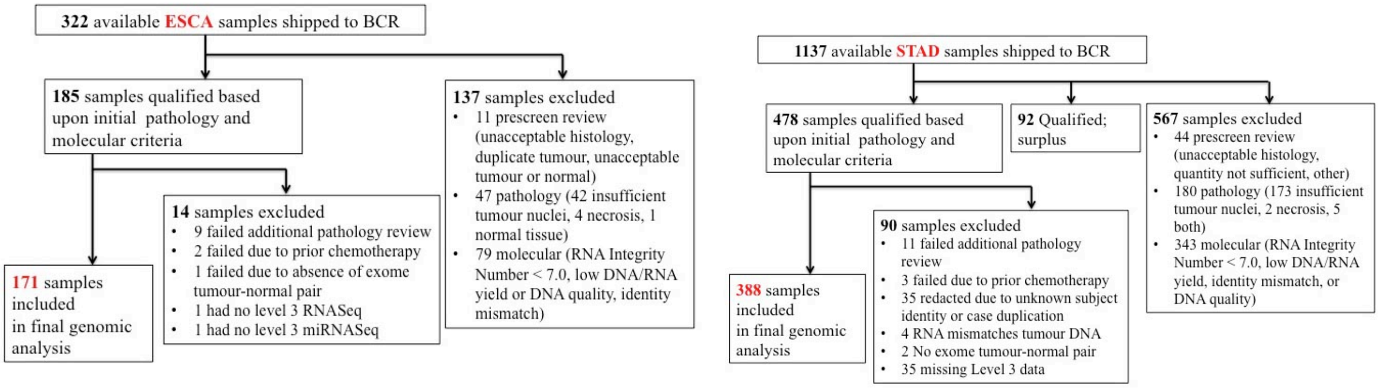

Figure S1.1. Tumour samples shipped to the Biospecimen Core Resource (BCR) were distributed as shown. ESCA and STAD are specimen identifiers for the Oesophagus and Stomach projects. As indicated above, 171 ESCA samples and 388 STAD samples were combined into a pool of 559 samples for analysis in these studies. From that pool, specimens were then evaluated by the Expert Pathologists' Committee (EPC). See Figure S1.2.

S1.2. Classification of Tumours.
Tumours occurring in the vicinity of the gastroesophageal junction (all putative oesophageal adenocarcinomas and any gastric tumours in the vicinity of the proximal stomach) were classified by the following criteria, based on pathology reports and independent review:

1. **Oesophagus** – Documented Barrett's oesophagus or intestinal metaplasia with a normal stomach or tumour grossly of the tubular oesophagus, or pattern of metastatic disease that was oesophageal and not stomach. (Cases of clear tubular origin were grouped with these oesophageal cases for subsequent analyses.)
2. **Probable Oesophagus** – none of the above, but with an epicenter in tubular oesophagus; no gastric intestinal metaplasia or normal stomach.
3. **Indeterminate**– no gastric intestinal metaplasia, tumour epicenter not indicated or not clear; stomach and oesophagus either normal or not indicated, pattern of nodal metastatic disease unclear or mixed.
4. **Probable gastric** – epicenter of tumour was in the proximal stomach; oesophagus was normal or not commented upon; there was chronic gastritis with or without intestinal metaplasia, or *Helicobacter pylori* was present; pattern of nodal metastatic disease was unclear (if present).
5. **Gastric** –oesophagus is normal or not commented upon; epicenter of the tumour in the stomach; presence of chronic gastritis with or without IM; gastric pattern of nodal metastatic disease (if present).

Independent of the original anatomical assignment by the TSS, the EPC determined that 36 gastroesophageal junction tumours could not be attributed to either a clear oesophageal or gastric origin. See Figure **S1.2** for distribution of tumours among anatomic sites.

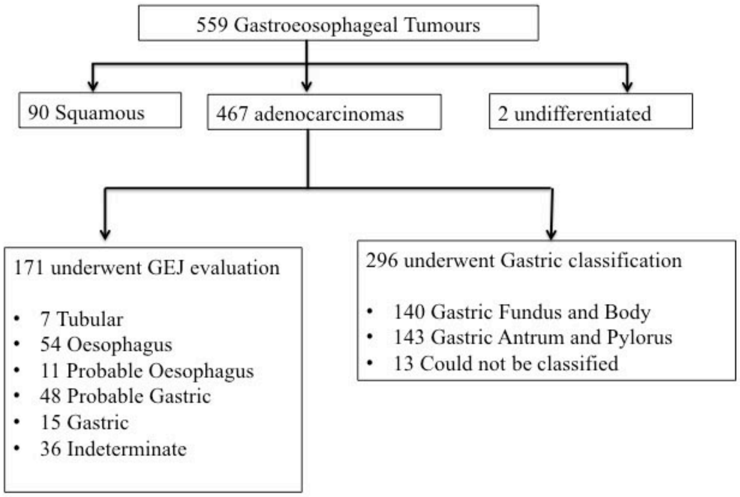

Histological and anatomic subclassification of gastroesophageal tumour samples.

Figure S1.2. Analysis of tumours of the GEJ.

S2. DNA Methylation

S2.1. Methylation Assay platform

The HM450 assay used in this study analyses the DNA methylation status of up to 482,421 CpG and 3,091 non-CpG (CpH) sites throughout the genome. It covers 99% of RefSeq genes with multiple probes per gene and 96% of CpG islands from the UCSC database, plus their flanking regions.

The DNA methylation score for each assayed CpG or CpH site is represented as a beta (β) value ($\beta = (M/(M+U))$ in which M and U indicate the mean methylated and unmethylated signal intensities for each assayed CpG or CpH, respectively. β-values range from zero to one, with scores of "0" indicating no DNA methylation and scores of "1" indicating complete DNA methylation. A detection *P* value accompanies each data point and compares the signal intensity difference between the analytical probes and a set of negative control probes on the array. Any data point with a corresponding *P* value greater than 0.05 is deemed not to be statistically significantly different from background and is thus masked as "NA" in the Level 3 data packages as described below. Further details on the Illumina Infinium DNA methylation assay technology have been described previously[2,3].

S2.2. Sample and data processing

We assessed the amount of bisulfite-converted DNA and completeness of bisulfite conversion using a panel of MethyLight-based quality control (QC) reactions as previously described[4]. All the TCGA samples passed our QC tests and entered the Infinium DNA methylation assay pipeline. Bisulfite-converted DNAs were whole-genome-amplified (WGA) and enzymatically fragmented prior to hybridisation to BeadChip arrays. BeadArrays were scanned using the Illumina iScan technology to produce IDAT files. Raw IDAT files for each sample were processed with the R/Bioconductor package methylumi. TCGA DNA methylation data packages were then generated using the EGC.tool*s* R package, which was developed internally and is publicly available on GitHub (https://github.com/uscepigenomecenter/EGC.tools).

S2.3. TCGA Data Packages

The data levels and the files contained in each data level package are described below and are present on the TCGA Data Portal website (http://tcga-data.nci.nih.gov/tcga/). As continuing updates of genomic databases and data archive revisions frequently become available, the data packages on TCGA Data Portal will be updated accordingly.

Level 1 data contain raw IDAT files (two per sample) as produced by the iScan system and as mapped by the Sample and Data Relationship Format (SDRF). These IDAT files were directly processed by the R/Bioconductor package methylumi. We provided a disease-mapping file (ESCA.mappings.csv for oesophageal cancer and STAD.mapping.csv for gastric cancer) in the AUX directory to facilitate this process. Level 2 data contain background-corrected methylated (M) and unmethylated (U) summary intensities as extracted by the R/Bioconductor package methylumi. Detection *P* values were computed as the minimum of the two values (one per allele) for the empirical cumulative density function of the negative control probes in the appropriate colour channel. Background correction was performed via normal-exponential deconvolution. Multiple-batch archives had the intensities in each of the two channels multiplicatively scaled to match a reference sample (sample with R/G ratio of the normalisation control probes closest to 1.0). Level 3 data contain β-value calculations with annotations for HGNC gene symbol, chromosome, and genomic coordinates (UCSC hg19, Feb 2009) for each targeted CpG/CpH site on the array. Probes having a common SNP (dbSNP build 135, Minor Allele Frequency >1%) within 10 bp of the interrogated CpG site or having an overlap with a repetitive element (as detected by

RepeatMasker and Tandem Repeat Finder based on UCSC hg19, Feb 2009) within 15 bp (from the interrogated CpG site) were masked as “NA” across all samples, and probes with a detection P value greater than 0.05 in a given sample were masked as “NA” on that array. Probes that were mapped to multiple sites in the human genome (UCSC hg19, Feb 2009) were annotated as “NA” for chromosome and 0 for the CpG/CpH coordinate.

The following data archives were used for the analyses described in this manuscript.

Oesophageal cancer:
jhu-usc.edu_ESCA.HumanMethylation450.Level_3.1.8.0
jhu-usc.edu_ESCA.HumanMethylation450.Level_3.2.8.0
jhu-usc.edu_ESCA.HumanMethylation450.Level_3.3.8.0
jhu-usc.edu_ESCA.HumanMethylation450.Level_3.4.8.0
jhu-usc.edu_ESCA.HumanMethylation450.Level_3.5.8.0
jhu-usc.edu_ESCA.HumanMethylation450.Level_3.6.8.0
jhu-usc.edu_ESCA.HumanMethylation450.Level_3.7.8.0
jhu-usc.edu_ESCA.HumanMethylation450.Level_3.8.8.0
jhu-usc.edu_ESCA.HumanMethylation450.Level_3.9.8.0
jhu-usc.edu_ESCA.HumanMethylation450.Level_3.10.8.0

Gastric adenocarcinoma:
jhu-usc.edu_STAD.HumanMethylation450.Level_3.1.10.0
jhu-usc.edu_STAD.HumanMethylation450.Level_3.2.10.0
jhu-usc.edu_STAD.HumanMethylation450.Level_3.3.10.0
jhu-usc.edu_STAD.HumanMethylation450.Level_3.4.10.0
jhu-usc.edu_STAD.HumanMethylation450.Level_3.5.10.0
jhu-usc.edu_STAD.HumanMethylation450.Level_3.6.10.0
jhu-usc.edu_STAD.HumanMethylation450.Level_3.7.10.0
jhu-usc.edu_STAD.HumanMethylation450.Level_3.8.10.0
jhu-usc.edu_STAD.HumanMethylation450.Level_3.9.10.0
jhu-usc.edu_STAD.HumanMethylation450.Level_3.10.10.0
jhu-usc.edu_STAD.HumanMethylation450.Level_3.11.10.0
jhu-usc.edu_STAD.HumanMethylation450.Level_3.12.10.0
jhu-usc.edu_STAD.HumanMethylation450.Level_3.13.10.0
jhu-usc.edu_STAD.HumanMethylation450.Level_3.14.10.0
jhu-usc.edu_STAD.HumanMethylation450.Level_3.15.10.0
jhu-usc.edu_STAD.HumanMethylation450.Level_3.16.10.0

S2.4. Unsupervised clustering analysis of DNA methylation data

We removed probes that had any “NA”-masked data points and probes that were designed for sequences on X and Y chromosomes.

To capture cancer-specific DNA hypermethylation events, we first selected CpG sites that were not methylated in normal tissue controls (mean β-value <0.2). To minimise the influence of variable tumour purity levels on a clustering result, we dichotomised the data using a β-value of >0.25 as a threshold for positive DNA methylation. The dichotomisation not only ameliorated the effect of tumour sample purity on the clustering, but

also removed a great portion of residual batch/platform effects that are mostly reflected in small variations near the two ends of the range of β-values. We also removed CpG sites that were methylated in leukocytes, a major source of contamination present in a tumour sample (mean β-value >0.2). We then performed consensus clustering with the dichotomised data on CpG sites that were methylated in at least 5% of the tumour samples. The optimal number of clusters was assessed based on 80% probe and tumour resampling over 1,000 iterations of hierarchical clustering for K=2,3,4,5,6 using the binary distance metric for clustering and Ward's method for linkage as implemented in the R/Bioconductor ConsensusClusterPlus package.

Heatmaps were generated based on the original β-values for a subset of hypermethylated CpG sites. The probes were displayed based on the order of unsupervised hierarchical clustering of the β-values using the Euclidean distance metric and Ward's linkage method.

S2.5. DNA hypermethylation frequency in 164 oesophageal tumours

We identified a set of 136,705 CpG sites that were unmethylated in adjacent oesophageal tissue samples (mean β-value <0.2) and leukocytes (mean β-value <0.2). We dichotomised the β-values in the tumours at 0.3. For each locus, tumours with a β-value of 0.3 or greater were designated as methylated, and tumours with a β-value of lower than 0.3 were designated as unmethylated. We then calculated the percentage of loci that were methylated among the loci investigated in each tumour.

S2.6. Identification of epigenetically silenced genes in GEA-CIN tumours

We first removed DNA methylation probes overlapping with SNPs, repeats or designed for sequences on X or Y chromosomes or non-CpG sites. The remaining probes were mapped against UCSC Genes using the GenomicFeatures R/Bioconductor package. Probes that were located in a promoter region (defined as the 3 kb region spanning from 1,500 bp upstream to 1,500 bp downstream of the transcription start sites) were identified. Level 3 mRNA expression data were log2 transformed [log2 (RPKM+1)] and used to assess the gene expression levels associated with DNA methylation changes. DNA methylation and gene expression data were merged by Entrez Gene IDs.

We removed the CpG sites that were methylated in normal tissues (mean β-value >0.2). We then dichotomised the DNA methylation data using a β-value of >0.3 as a threshold for positive DNA methylation, and further eliminated CpG sites methylated in fewer than 3% of the tumour samples. For each probe/gene pair, we applied the following algorithm: 1) Organise the tumours as either methylated (β ≥0.3) or unmethylated (β <0.3); 2) Compute the mean expression in the methylated and unmethylated groups; 3) Compute the standard deviation of the expression in the unmethylated group. We then selected probes for which the mean expression in the methylated group was lower than 1.64 standard deviations of the mean expression in the unmethylated group. We labeled each individual tumour sample as epigenetically silenced for a specific probe/gene pair selected from above if: a) it belonged to the methylated group and b) the expression of the corresponding gene was lower than the mean of the unmethylated group of samples. If there were multiple probes associated with the same gene, a sample identified as epigenetically silenced at more than half the probes for the corresponding gene was also labeled as epigenetically silenced at the gene level. The complete list of 66 genes identified as epigenetically silenced is cluster 1 of the GEA-CIN methylation groups and is provided in Supplementary data Table 6

S2.7. Statistics

Statistical analysis and data visualisation were performed using the R/Bioconductor software packages (http://www.bioconductor.org).

S3. DNA Sequencing

S3.1. Multi-center analysis of somatic mutations in exome sequencing

Mutation calling of the whole exome sequencing data was completed in parallel at four genomic analysis centers:

1) *Broad Institute*: The Broad Institute team used the MuTect algorithm to generate somatic mutation calls, which were subsequently filtered to remove any spurious calls due to shearing-induced generation of 8-oxoguanine[5]. Indels were identified using the indel locator algorithm as previously described[6]. Details and tools are available at www.broadinstitute.org/cancer/cga.

2) *University of California and Santa Cruz:* The RADIA software[7] for identification of somatic mutations is available at https://github.com/aradenbaugh/radia/. Inclusion of RNA-Seq data in RADIA increases the power to detect somatic mutations at low DNA allelic frequencies.

3) British Columbia Cancer Genome Agency: Mutation calling was performed using the Strelka tool. Strelka[8] parameters were set to default, with the exception of "isSkipDepthFilters", which was set to 1 in order to omit depth filtration, given the higher coverage in exome datasets. Pairs of libraries (n=184) were analysed. When a blood sample was available, it served as the matched normal specimen; otherwise, the matched normal tissue was used. The variants were subsequently annotated using SnpEff[9], and the COSMIC (v61)[10] and dbSNP (v137)[11] databases.

4) *Washington University in St. Louis*: We detected somatic SNVs using Samtools1 v0.1.16 (samtools pileup –cv -A -B), SomaticSniper2 v1.0.4 (bam-somaticsniper -F vcf -G -L -q 1 -Q 15), Strelka3 v0.4.6.2 (with default parameters except for setting isSkipDepthFilters = 1), and VarScan4 v2.2.6 (--min-coverage 3 --min-var-freq 0.08 --p-value 0.10 --somatic-p-value 0.05 --strand-filter 1). We detected indels using the GATK5 1.0.5336 (-T IndelGenotyperV2 --somatic --window_size 300 -et NO_ET), retaining only those which were called as Somatic, Pindel6 v0.2.2 (-w 10; with a config file generated to pass both tumour and normal BAM files set to an insert size of 400), Strelka3 v0.4.6.2 (with default parameters except for setting isSkipDepthFilters = 1), and VarScan4 v2.2.6 (--min-coverage 3 --min-var-freq 0.08 --p-value 0.10 --somatic-p-value 0.05 --strand-filter 1).

S3.2. Mutation Signature Analysis.

We used the Bayesian non-negative matrix factorization algorithm (BayesNMF)[12,13] to infer the number (K) of mutational signatures (characteristic mutational patterns) and their sample-specific contributions. The common classification of single nucleotide variants is based on six base substitutions within the tri-nucleotide sequence context including the bases immediately 5' and 3' to each mutated base. Six base substitutions (C>A, C>G, C>T, T>A, T>C, and T>G) with 16 possible combinations of neighboring bases results in 96 possible mutation types within trinucleotide regions. Thus the input data for the mutation signature discovery is given as 96 by *M* mutation matrix (M= # of sample). The mutation count matrix was taken as an input for the BayesNMF and factored into two matrices, **W'** (96 by K) and **H'** (K by M), approximating **X** by **W'H'**. To enumerate the number of mutations associated with each mutation signature, we performed a scaling transformation, $\mathbf{X} \sim \mathbf{W'H'} = \mathbf{WH}$, $\mathbf{W} = \mathbf{W'U}^{-1}$ and $\mathbf{H} = \mathbf{UH'}$, where **U** is a K by K diagonal matrix with the element corresponding to the 1-norm of column vectors of **W'**, resulting in the final signature matrix **W** and the activity matrix **H**.

All 20 BayesNMF runs for the SCC subgroup converged to the 3-signature solution (K=3), yielding APOBEC (corresponding to COSMIC signature 2 and COSMIC signature 13), Aging (COSMIC signature 1), and "Unknown". In contrast to two other signatures, the unknown signature was characterised by a dominant C>A mutation signature, and its mutation pattern showed a similarity with several COSMIC signatures, including COSMIC 4 (smoking), 24 (Aflatoxin B1), and 29 (tobacco-chewing habits). We used the cosine similarity to compare our three signatures with thirty reported COSMIC signatures (http://cancer.sanger.ac.uk/cosmic/signatures).

Note that the *k*th column vector of **W** (**w_k**) represents a normalised mutability of 96 tri-nucleotide mutation contexts in the *k*th signature, and the *k*th row vector of **H** (**h_k**) dictates the estimation of mutations associated with the *k*th signature across samples. In downstream signature enrichment analysis, we compared both the number of mutations and the normalised fraction of mutations associated with each signature.

S3.3 Additional Somatic Genomic Analyses.

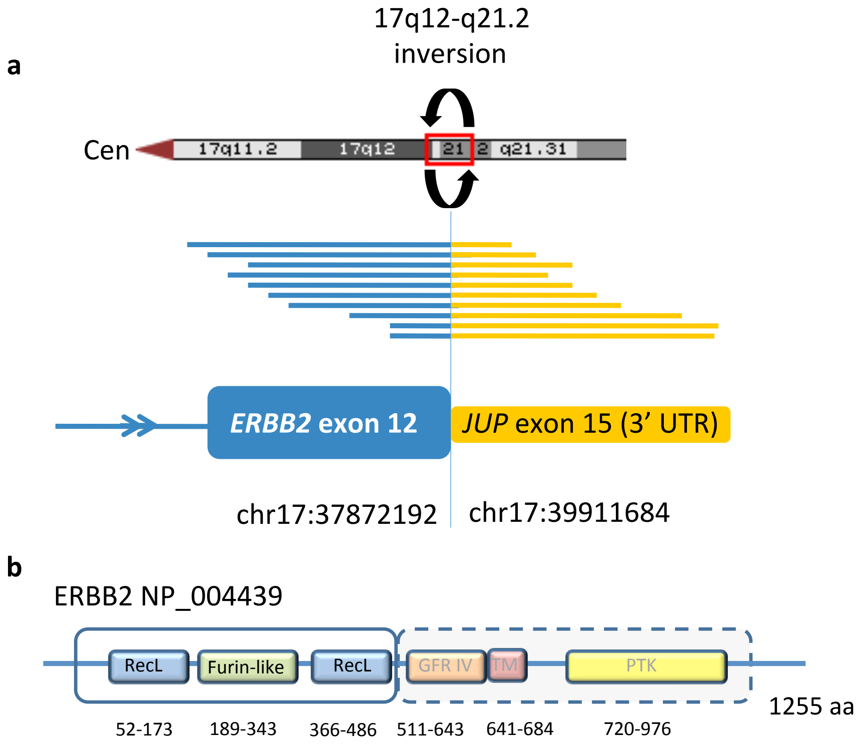

Figure S3.1: Recurrent *ERBB2-JUP* gene fusion in six TCGA oesophageal adenocarcinoma cases. a, A 2-Mb inversion event at 17q12-21.2 created a gene fusion juxtaposing *ERBB2* exon 12 (erb-b2 receptor tyrosine kinase 2, NM_004448) to the 3'UTR (exon 15) of *JUP* (junction plakoglobin, transcript variant 2). Trans-ABySS assembly and structural variant detection in RNA sequence reads revealed six cases harbouring the same fusion transcript. Chromosome 17 breakpoint coordinates are from the GRCh37/hg19 human genome assembly. b, The fusion breakpoint in ERBB2 removes amino acids 505 to 1255 of ERBB2, including the growth factor domain (aa 511-643), transmembrane domain (aa 641-684) and the protein kinase domain (aa 720-976).

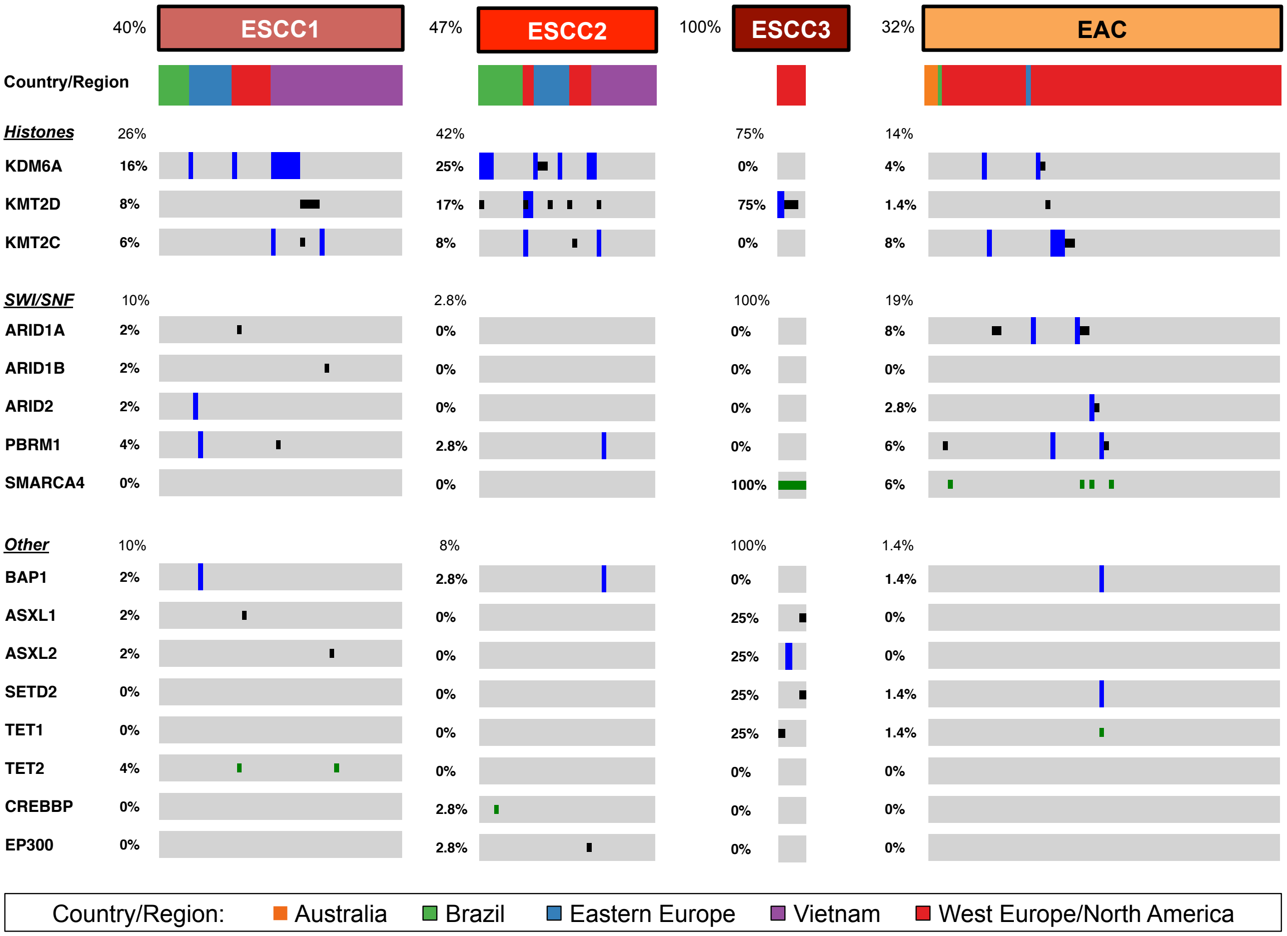

Figure S3.2. Mutations of Genes Encoding Chromatin Modifying Enzymes in Oeosophageal Cancer. Somatic events in the three classes of oesophageal squamous cell carcinoma and in oesophageal adenocarcinoma are shown. Blue boxes indicate genomic deletion, black boxes are truncating mutations and green boxes denote missense mutations of distinct chromatin modifying factors. Only missense mutations at recurrent hotspots or those previously reported in the COSMIC repository were included.

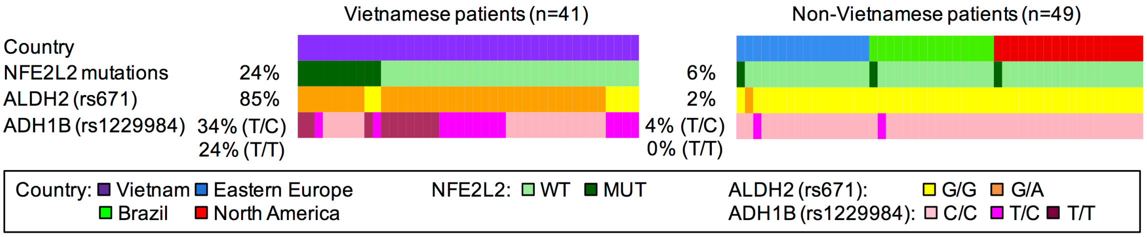

Figure S3.3. Regional differences in frequencies of *NFE2L2* somatic mutations and metabolic germline variants with potential genotoxic effects in the ESCC cohort. The prevalence of *NFE2L2* somatic mutations was higher in Vietnamese patients than in patients from outside East Asia. Similarly, non-synonymous SNPs in aldehyde dehydrogenase (*ALDH2*) and alcohol dehydrogenase (*ADH1B*) were more frequent in the Vietnamese population and affected every East Asian patient in our study. The genotypes for specific alleles in patients with or without key somatic alterations in oxidative response genes are shown.

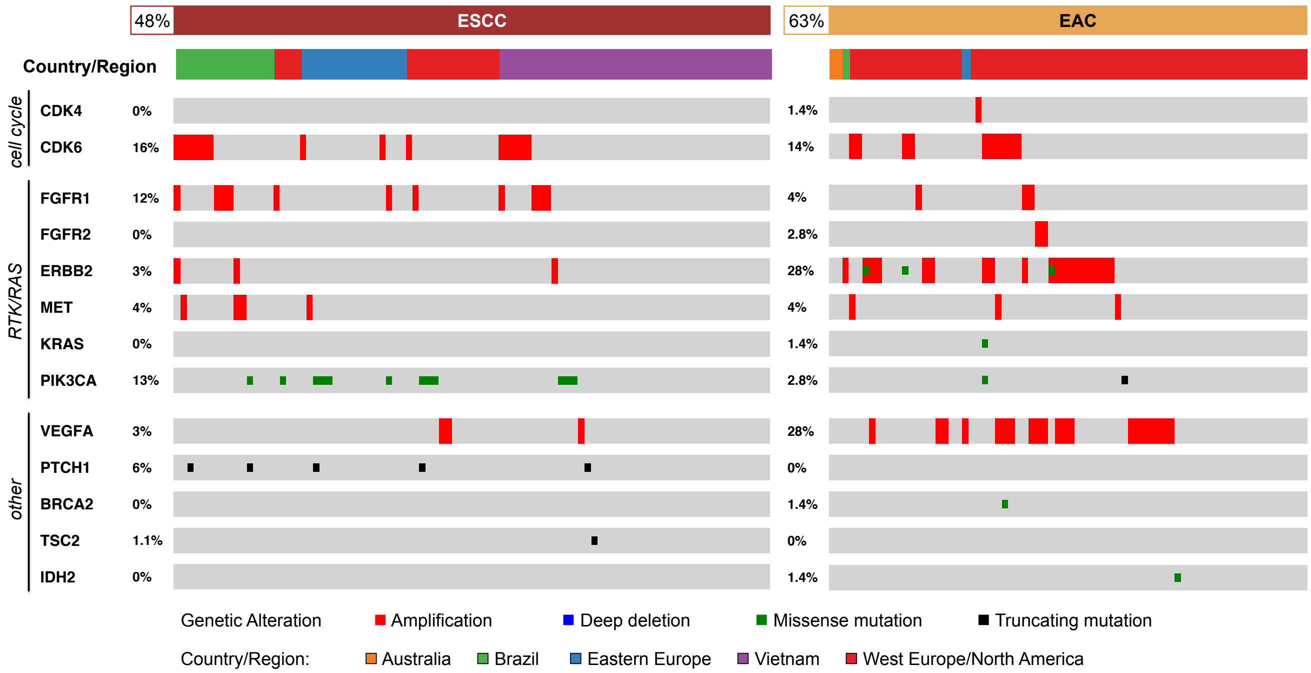

GENE	ALTERATION	LEVEL*	DRUG	CANCER TYPE INDICATION
ERBB2	Amplification	SoC	Trastuzumab	Stomach adenocarcinoma
	Mutation (V777L,S310F)	Inv	Neratinib	Breast cancer
BRCA2	Mutation (S206C)	Inv	Olaparib	Ovarian cancer
TSC2	Mutation (E409*)	Inv	Everolimus	Central nervous system cancer
CDK4/6	Amplification	Inv	Palbociclib	Soft Tissue Sarcoma
MET	Amplification	Inv	Crizotinib	Non-small Cell Lung Cancer
KRAS	Mutation (Q61H)	Inv	Binimetinib+alpelisib	Ovarian cancer
			Selumetinib+Docetaxel	Non-small Cell Lung Cancer
PIK3CA	Mutation (E545K, G118D, H1047R/L, M1043I, K111N)	Inv	BYL-719	Breast cancer
PTCH1	Mutation (any truncating mutation)	Inv	Sonidegib	Embryonal Tumour, Skin Cancer, Non-melanoma
FGFR1	Amplification	Inv	AZD4547	Non-small Cell Lung Cancer
			Dovitinib	Breast Cancer
FGFR2	Amplification	Inv	Dovitinib	Breast Cancer
IDH2	Mutation (R140Q)	Inv	AG-221	All liquid tumours
VEGFA	Amplification	Inv	Ramucirumab	Gastroesophageal junction adenocarcinoma

* Level of evidence is either standard-of-care (SoC) or investigational (I).

Figure S3.4. Putative Therapeutic Targets and Biomarkers in Oesophageal Cancer.
Summary of clinically actionable somatic alterations in our cohort of oesophageal cancer patients. Top panel shows occurrence of clinically actionable somatic alterations, divided by histology and region of origin. Bottom panel provides additional details about each actionable alteration, including level of evidence, related drug, and specific cancer type(s) for which sensitivity to the drug is clinically proven or currently undergoing clinical trials. Annotations were obtained from OncoKB, which is a publicly available database for precision medicine curated by researchers at Memorial Sloan-Kettering Cancer Center.

S4. Reverse Phase Protein Arrays

S4.1. Methods

Protein was extracted from human tumours using RPPA lysis buffer (1% Triton X-100, 50 nmol/L Hepes (pH 7.4), 150 nmol/L NaCl, 1.5 nmol/L $MgCl_2$, 1 mmol/L EGTA, 100 nmol/L NaF, 10 nmol/L NaPPi, 10% glycerol, 1 nmol/L phenylmethylsulfonyl fluoride, 1 nmol/L Na_3VO_4, and aprotinin 10 μg/mL), and RPPA was performed as described previously[1,14-17]. Lysis buffer was used to lyse frozen tumours by Precellys homogenisation. Tumour lysates were adjusted to 1 μg/μL concentration as assessed by bicinchoninic acid assay (BCA) and boiled with 1% SDS. Tumour lysates were manually diluted in fivefold serial dilutions with lysis buffer. An Aushon Biosystems 2470 arrayer (Burlington, MA) printed 1,056 samples on nitrocellulose-coated slides (Grace Bio-Labs). Slides were probed with 187 validated primary antibodies followed by corresponding secondary antibodies (Goat anti-Rabbit IgG, Goat anti-Mouse IgG or Rabbit anti-Goat IgG). Signal was captured using a DakoCytomation-catalysed system and DAB colorimetric reaction. Slides were scanned in a CanoScan 9000F scanner. Spot intensities were analysed and quantified using Arrapro (http://www.mediacy.com/index.aspx?page=ArrayPro), to generate spot signal intensities (Level 1 data). The software SuperCurveGUI[16,18], available at http://bioinformatics.mdanderson.org/Software/supercurve/) was used to estimate the EC50 values of the proteins in each dilution series (in log2 scale). Briefly, a fitted curve ("supercurve") was plotted with the signal intensities on the Y-axis and the relative log2 concentration of each protein on the X-axis, using the non-parametric, monotone increasing B-spline model[14]. During the process, the raw spot intensity data were adjusted to correct spatial bias before model fitting. A quality control (QC) metric[18] was returned for each slide to help determine the quality of the slide: if the score was less than 0.8 on a 0-1 scale, the slide was omitted. In most cases, the staining was repeated to obtain a high quality score. If more than one slide was stained with an antibody, the slide with the highest QC score was used for analysis (Level 2 data). Protein measurements were corrected for loading as described[16,18,19] using median centering across antibodies (level 3 data). In total, 187 antibodies and 433 samples were used; 113 oesophageal cancer (ESCA) and 320 stomach adenocarcinoma (STAD) samples. Final selection of antibodies was determined by the availability of high quality antibodies that consistently passed a strict validation process, as previously described[20]. These antibodies were assessed for specificity, quantification and sensitivity (dynamic range) in their application for protein extracts from cultured cells or tumour tissue. Antibodies were labeled as 'validated' or 'use with caution', based on degree of validation by criteria previously described[20].

Raw data (level 1), SuperCurve nonparameteric model-fitted data on a single array (level 2), and loading-corrected data (level 3) were deposited at TCGA Data Coordinating Center (DCC).

S4.2. Data normalisation

We performed median centering across all the antibodies for each sample to correct for sample loading differences. Those differences arise because protein concentrations are not uniformly distributed per unit volume. That condition may be due to several factors, such as differences in protein concentrations of large and small cells, differences in the amount of proteins per cell, or heterogeneity of the cells comprising the samples. By observing the expression levels across many different proteins in a sample, we can estimate differences in the total amount of protein in that sample vs. other samples. Subtracting the median protein expression level forces the median value to become zero, allowing us to compare protein expressions across samples.

Surprisingly, processing similar sets of samples on different slides with the same antibody may result in datasets that have very different means and variances. Neely et al.[21] processed clinically similar acute lymphoblastic leukemia samples in two batches and observed differences in their protein data distributions. There were

additive and multiplicative effects in the data that could not be accounted for by biological or sample-loading differences. We observed similar effects when we compared the two batches of tumour protein expression data. A new algorithm, replicates-based normalisation (RBN), was therefore developed using replicate samples run across multiple batches, to adjust the data for batch effects. The underlying hypothesis is that any observed variation between replicates in different batches is primarily due to linear batch effects plus a component due to random noise. Given a sufficiently large number of replicates, the random noise is expected to cancel out (mean=zero, by definition). Remaining differences are treated as systematic batch effects. We can compute those effects for each antibody and subtract them out. Many samples were run in both batches. One batch was arbitrarily designated the "anchor" batch and was to remain unchanged. We then computed the means and standard deviations of the common samples in the anchor batch, as well as the other batch. The difference between the means of each antibody in the two batches and the ratio of the standard deviations provided an estimate of the systematic effects between the batches for that antibody (both location-wise and scale-wise). Each data point in the non-anchor batch was adjusted by subtracting the difference in means and multiplying by the inverse ratio of the standard deviations to cancel out those systematic differences. Our normalisation procedure significantly reduced technical effects, thereby allowing us to merge the datasets from different batches.

S4.3. Unsupervised hierarchical clustering analysis

We used ConsensusClusterPlus package in R v2.13.2 to identify robust subtypes for distinct sample sets evaluated in this analysis based on protein expression[22] . The consensus clusters were obtained from 1,000 resampling iterations of the hierarchical clustering, by randomly selecting a fraction of the samples and of the protein features.

Unsupervised hierarchical clustering analyses were carried out with two data sets. The first analysis with all oesophageal tumours (n = 113) revealed three robust RPPA clusters (**Extended Data Figure 1**). These three RPPA subtypes were significantly correlated with histological subtypes of oesophageal cancer (EAC and ESCC), $p=1.1 \times 10^{-7}$). RPPA cluster 1 (E1) was associated with EAC and expressed high levels of Claudin7 and TIGAR. Cluster 2 (E2) was associated with low expression of BRAF, MTOR, CDH1, and ATM, which play roles in cell proliferation and cell adhesion, suggesting that tumours in cluster 2 might have increased potential of invasion and metastasis (i.e., an EMT phenotype). Cluster 3 (E3) was characterised by high expression of fibronectin and PAI1. Analysis of all CIN subtype tumours (including both gastric and oesophageal adenocarcinoma) revealed three clusters that were different from the previous two analyses (**Extended Data Figure 9**). In particular, the C3 cluster was associated with higher expression of RICTOR, CAV1, and MYH11, likely reflecting activation of stromal cells in the tumour microenvironment.

S5. Microbiome analysis

S5.1. Methods of microbial detection in RNA-Seq and whole exome data

BioBloomTools (BBT, v1.2.4.b1) is a Bloom filter-based method for rapidly classifying RNA-seq or DNA-seq read sequences[1]. We generated 43 filters from 'complete' NCBI genome reference sequences of bacteria, viruses, fungi and protozoa, using 25-bp k-mers and a false positive rate of 0.02. We ran BBT in paired-end mode with a sliding window to screen FASTQ files from RNA-seq libraries (75-bp paired end reads and 38 GAIIx samples with 50-bp paired end reads), and whole exome libraries (49-bp PE reads). In a single-pass scan for each library, BBT categorised each read pair as matching the human filter, matching a unique microbial filter, matching more than one filter (multi-match), or matching neither human nor microbe (no-match). For each filter, we then calculated a reads-per-million (RPM) abundance metric as follows:

$$Abundance\ metric = \left(\frac{\#\,reads\,mapped\,to\,the\,microbe}{\#\,reads\,mapped\,to\,human\,in\,the\,sample} * 10^6 \right)$$

Samples with at least one read-pair classified as Epstein Barr Virus (EBV, also called human herpes virus 4, HHV4) by *BioBloom* in RNA-Seq data were submitted to EBV gene-expression analysis. For this analysis, BWA-0.5.7 was used to align reads to a custom-created reference based on the NCBI EBV type 1 complete genome and gene annotations (NC_007605.1). Reads with an alignment spanning exon-exon junctions were then transformed into large gapped genomic alignments using JAGuaR. Reads with a mapping quality of 10 or greater were included in the gene expression quantification analysis. Results were normalised to reads per kilobase of exon per million reads mapped to the EBV transcriptome.

S5.2. Microbial detection in miRNA-Seq data

We quantified levels of microbial content in miRNA sequence data in 604 gastric and oesophageal tumour and normal samples using methods described previously[23,24].

S5.3. Methods of microbial detection in RNA-Seq, whole genome and whole exome data

The PathSeq algorithm[25] was used to perform computational subtraction of human reads, followed by alignment of residual reads to human reference genomes and microbial reference genomes (which include bacterial, viral, archaeal, and fungal sequences - downloaded from NCBI in June, 2012). These alignments resulted in the identification of reads mapping to the EBV and HPV genome in whole genome sequencing (WGS) and whole exome sequencing (WES) data.

In brief, human reads were subtracted by first mapping reads to a database of human genomes (downloaded from NCBI in November 2011) using BWA[26] (Release 0.6.1, default settings), Megablast (Release 2.2.25, cut-off E-value 10^{-7}, word ss 16) and Blastn[27] (Release 2.2.25, cut-off E-value 10^{-7}, word size 7, nucleotide match reward 1, nucleotide mismatch score -3, gap open cost 5, gap extension cost 2). Only sequences with perfect or near perfect matches to the human genome were removed in the subtraction process. In addition, low complexity and highly repetitive reads were removed using Repeat Masker (version open-3.3.0, libraries dated 2011-04-19; http://www.repeatmasker.org).

To identify EBV and HPV reads, the residual reads were aligned with Megablast to a database of microbial and human reference genomes. Raw read counts were calculated using the reads that were mapped to the viral genome with at least 90% identity and 90% query coverage.

Using the raw read counts, the abundance metric or normalised read count of a given microbe in a sample was calculated as follows:

$$Abundance\ metric = \left(\frac{\#\, reads\, mapped\, to\, the\, microbe}{\left(\frac{\#\, reads\, mapped\, to\, human\, in\, the\, sample}{Average\, \#\, reads\, mapped\, to\, human\, in\, the\, sample\, cohort} \right) * \left(\frac{Genome\, size\, of\ the\, microbe}{Average\ \ genome\, size\, of\ the\, microbes\, in\, that\, kingdom} \right)} \right)$$

Relative abundance in a given sample was calculated as abundance metric of taxa divided by the total abundance metric at kingdom level of the sample. Samples were considered to be EBV positive if the abundance metric exceeded 1000 by WGS or 100 by WES. HPV read count levels were compared between ESCC subtypes in **Figure 3e**.

S6. Survival Analysis

We evaluated patient survival (overall survival) for the ESCC1 and ESCC2 groups of oesophageal squamous cell carcinoma by both plotting Kaplan-Meier survival curves and by calculating 95% confidence intervals for Cox proportional hazards ratio as shown below in **Figure S6.1**. There was no significant difference (p=0.93) between survivals in the squamous iCluster groups based on either approach. The covariates used for the Cox model were age (modeled with a spline), size (from T-stage), positive lymph nodes (from N-stage), grade, gender, and *TP53* mutation. Survival values were censored at 3 years. Given the small numbers of tumours in ESCC3, we excluded these from survival analysis.

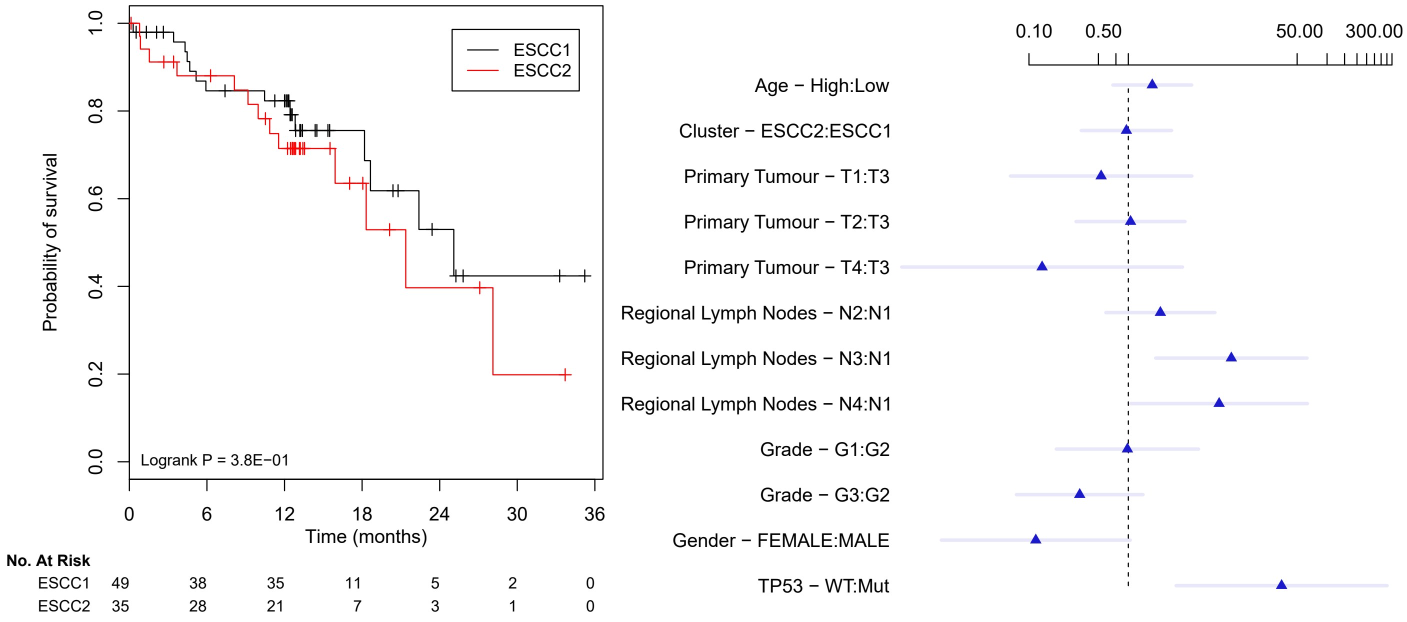

Figure S6.1: Survival analysis of oesophageal squamous cell carcinoma clusters. At left are the Kaplan-Meier survival curves for ESCC1 and ESCC2, showing no significant difference. At right are survival estimates with Cox-proportional hazards for ESCC1 and ESCC2 with covariates. In the right panel, the age comparison was High (61.21 years and older) compared to Low (younger than 51.56 years).

S7. Integrative clustering

S7.1.

Integrative clustering with multiple kernel learning

Integrative clustering was performed using multiple kernel learning with the mRNA, miRNA, copy-number and methylation datasets to evaluate the GEA-CIN group of tumours. The kernelised variant of K-means, proposed by Girolami[28] transforms the original input space to a kernel that essentially represents the similarity between the samples and applies the regular k-means algorithm to the k largest eigenvalues from the principal component analysis of the kernel. For this clustering analysis, we used an integrative approach[29,30] which combines Multiple Kernel Learning (MKL) paradigm with this Kernel K-means approach, enabling the clustering of multiple different molecular datasets. MKL approaches have proved valuable in the integration of distinct molecular datatypes[31-33]. The MKL K-means approach typically exhibits good performance in datasets with more features than samples and also allows for the modelling of non-linear relationships amongst features both within and between datatypes.

Data preprocessing: The dimensionality of the segmented copy-number data was reduced using CNRegions from iClusterPlus package. For the DNA methylation data, we combined B-values derived from the 450K and 27K platforms using the MergeMethylationData function from the TCGA Assembler package[34] and imputed missing values. mRNA and miRNA data were log-normalised.

Algorithm parameterisation: We employed the Matlab implementation of Gönen et al.[30] and the kernel adjustment and normalisation from SimpleMKL[35]. For each datatype, we used a 3 Gaussian kernel configuration with (0.95,1,1.05)*sqrt(number of features of each dataset).

Model selection: In order to ensure the robustness of the clusters, we performed 100 external bootstraps using 95% of the samples and an internal k-means 10-times bootstrap with random centroid initialisation. Using several information criteria (variation of information, Rand index, Silhouette width and Dunn index) to select the appropriate number of clusters k between 2 and 12, we found that the "elbow" with highest difference in variation and the Rand Index fell between 7 and 8, so we selected 7 clusters. We show the 7-clusters result (Extended Data Figure 9) and the mapping to other cluster assignments and clinical features.

Integrative clustering using iCluster

iCluster[36] generated a single integrated cluster assignment by a joint multivariate regression of multiple data types with respect to a set of common latent variables that represent the underlying tumour subtypes. The optimal number of clusters was determined using the Bayesian Information Criterion (BIC). iCluster analysis was also performed on the squamous cell carcinoma samples alone (n=90) to discover distinct molecular subtypes of ESCC, using a similar procedure.

Data were pre-processed as follows: Copy-number alteration data were derived from data segmented using the Circular Binary Segmentation (CBS) from the Affymetrix SNP6.0 platform, and further reduced to a set of 1155 non-redundant regions as described in ref[37]. For the methylation data (Illumina Infinium 450k arrays), Median Absolute Deviation (MAD) was used to select the top 1000 most variable CpG sites after a beta-mixture quantile normalisation using the Bioconductor package wateRmelon. Methylation probes with >20% missing

data and probes including SNPs or located on sex chromosomes were removed. For mRNA and miRNA sequence data, lowly expressed genes were excluded based on median-normalised counts, and variance filtering left 1223 mRNAs and 175 miRNAs remaining for clustering. mRNA and miRNA expression features were log2 transformed, normalised and scaled before submitting to iCluster. For the ESCC iCluster analysis, a similar data processing procedure was used, resulting in 1352 copy-number regions, 1000 most variable CpG sites, 1152 mRNA and 169 miRNA features based on MAD filtering.

Integrative clustering using COCA

Clustering of Cluster Assignments (COCA), or integrative clustering by platform-specific subtypes, was performed as described in Suppl S10.2 of Ref[1]. The method identifies shared molecular patterns among tumour samples based on the clusters obtained from individual platforms as described above: gene expression, miRNA expression, copy number and DNA methylation. The starting point is the construction of a binary matrix, in which columns represent samples, and rows correspond to each of the possible platform-specific cluster assignments. Each element of the matrix indicates whether a sample was a member of the platform-specific cluster corresponding to that row (1) or not (0). Samples are included in the analysis only if they have cluster assignments for all four platforms. To compare two samples, the method uses a distance that is based on counting the co-occurrence of their platform-specific cluster assignments, but also includes a weight to compensate for differences in the number of clusters available for each platform. Platform-specific clusters (rows) are also compared, by applying Fisher's exact test to the contingency table for sample membership, and transforming the resulting *p*-value to the negative of its logarithm. COCA was performed for the GEA set (**Extended Data Figure 8**) and for GEA-CIN (**Extended Data Figure** 10).

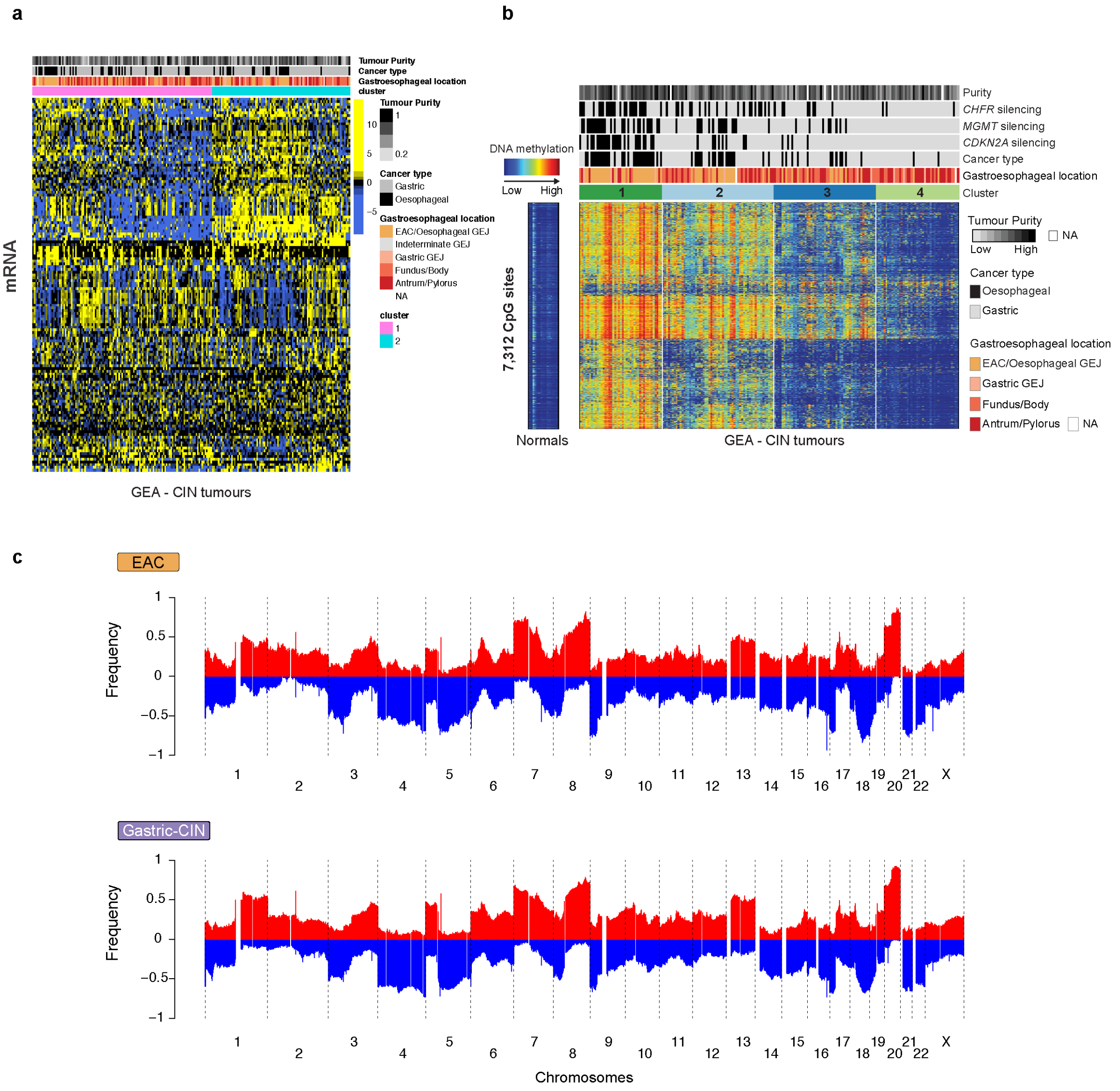

Supplemental Figure 7.1: Comparison of Gastroesophageal-CIN Adenocarcinomas Following Exclusion of Cases with Indefinite origin. Demonstrated in this figure is a comparison of features from mRNA expression (a), DNA methylation (b) and somatic copy-number (c) for CIN-GEA tumours following the removal of all cases with indeterminate location or those deemed probable oesophagus or probable gastric in origin. Panels a and b demonstrate the manner of clustering of the gastric and oesophageal GEA-CIN tumours for gene expression and methylation, respectively. Panel c demonstrates the similarity in SCNA profiles of these groups of tumours.

References

1. The Cancer Genome Atlas Research Network. Comprehensive molecular characterization of gastric adenocarcinoma. *Nature* **513**, 202-9 (2014).
2. Bibikova, M. *et al.* Genome-wide DNA methylation profiling using Infinium(R) assay. *Epigenomics* **1**, 177-200 (2009).
3. Bibikova, M. *et al.* High density DNA methylation array with single CpG site resolution. *Genomics* **98**, 288-95 (2011).
4. Campan, M., Weisenberger, D.J., Trinh, B. & Laird, P.W. MethyLight. *Methods Mol Biol* **507**, 325-37 (2009).
5. Costello, M. *et al.* Discovery and characterization of artifactual mutations in deep coverage targeted capture sequencing data due to oxidative DNA damage during sample preparation. *Nucleic Acids Res* **41**, e67 (2013).
6. Chapman, M.A. *et al.* Initial genome sequencing and analysis of multiple myeloma. *Nature* **471**, 467-72 (2011).
7. Radenbaugh, A.J. *et al.* RADIA: RNA and DNA integrated analysis for somatic mutation detection. *PLoS One* **9**, e111516 (2014).
8. Saunders, C.T. *et al.* Strelka: accurate somatic small-variant calling from sequenced tumor-normal sample pairs. *Bioinformatics* **28**, 1811-7 (2012).
9. Cingolani, P. *et al.* A program for annotating and predicting the effects of single nucleotide polymorphisms, SnpEff: SNPs in the genome of Drosophila melanogaster strain w1118; iso-2; iso-3. *Fly (Austin)* **6**, 80-92 (2012).
10. Forbes, S.A. *et al.* COSMIC (the Catalogue of Somatic Mutations in Cancer): a resource to investigate acquired mutations in human cancer. *Nucleic Acids Res* **38**, D652-7 (2010).
11. Smigielski, E.M., Sirotkin, K., Ward, M. & Sherry, S.T. dbSNP: a database of single nucleotide polymorphisms. *Nucleic Acids Res* **28**, 352-5 (2000).
12. Tan, V.Y. & Fevotte, C. Automatic relevance determination in nonnegative matrix factorization with the beta-divergence. *IEEE Trans Pattern Anal Mach Intell* **35**, 1592-605 (2013).
13. Kasar, S. *et al.* Whole-genome sequencing reveals activation-induced cytidine deaminase signatures during indolent chronic lymphocytic leukaemia evolution. *Nat Commun* **6**, 8866 (2015).
14. Tibes, R. *et al.* Reverse phase protein array: validation of a novel proteomic technology and utility for analysis of primary leukemia specimens and hematopoietic stem cells. *Mol Cancer Ther* **5**, 2512-21 (2006).
15. Liang, J. *et al.* The energy sensing LKB1-AMPK pathway regulates p27(kip1) phosphorylation mediating the decision to enter autophagy or apoptosis. *Nat Cell Biol* **9**, 218-24 (2007).
16. Hu, J. *et al.* Non-parametric quantification of protein lysate arrays. *Bioinformatics* **23**, 1986-94 (2007).
17. Hennessy, B.T. *et al.* Pharmacodynamic markers of perifosine efficacy. *Clin Cancer Res* **13**, 7421-31 (2007).
18. Coombes, K. *et al.* SuperCurve: SuperCurve Package. *R package version* **1.4.1**(2011).
19. Gonzalez-Angulo, A.M. *et al.* Functional proteomics can define prognosis and predict pathologic complete response in patients with breast cancer. *Clin Proteomics* **8**, 11 (2011).

20. Hennessy, B.T. *et al.* A Technical Assessment of the Utility of Reverse Phase Protein Arrays for the Study of the Functional Proteome in Non-microdissected Human Breast Cancers. *Clin Proteomics* **6**, 129-51 (2010).
21. Neeley, E.S., Kornblau, S.M., Coombes, K.R. & Baggerly, K.A. Variable slope normalization of reverse phase protein arrays. *Bioinformatics* **25**, 1384-9 (2009).
22. Wilkerson, M.D. & Hayes, D.N. ConsensusClusterPlus: a class discovery tool with confidence assessments and item tracking. *Bioinformatics* **26**, 1572-3 (2010).
23. The Cancer Genome Atlas Research Network. Comprehensive molecular portraits of human breast tumours. *Nature* **490**, 61-70 (2012).
24. Chu, A. *et al.* Large-scale profiling of microRNAs for The Cancer Genome Atlas. *Nucleic Acids Res* (2015).
25. Kostic, A.D. *et al.* PathSeq: software to identify or discover microbes by deep sequencing of human tissue. *Nat Biotechnol* **29**, 393-6 (2011).
26. Li, H. & Durbin, R. Fast and accurate short read alignment with Burrows-Wheeler transform. *Bioinformatics* **25**, 1754-60 (2009).
27. Altschul, S.F. *et al.* Gapped BLAST and PSI-BLAST: a new generation of protein database search programs. *Nucleic Acids Res* **25**, 3389-402 (1997).
28. Girolami, M. Mercer kernel-based clustering in feature space. *IEEE Trans Neural Netw* **13**, 780-4 (2002).
29. Yu, S. *et al.* Optimized data fusion for kernel k-means clustering. *IEEE Trans Pattern Anal Mach Intell* **34**, 1031-9 (2012).
30. Gönen, M. & Margolin, A.A. Localized data fusion for kernel k-means clustering with application to cancer biology. *Advances in Neural Information Processing Systems* **NIPS2014_5236**, 1305-1313 (2014).
31. Daemen, A. *et al.* A kernel-based integration of genome-wide data for clinical decision support. *Genome Med* **1**, 39 (2009).
32. Yu, S. *et al.* L2-norm multiple kernel learning and its application to biomedical data fusion. *BMC Bioinformatics* **11**, 309 (2010).
33. Seoane, J.A., Day, I.N., Gaunt, T.R. & Campbell, C. A pathway-based data integration framework for prediction of disease progression. *Bioinformatics* **30**, 838-45 (2014).
34. Zhu, Y., Qiu, P. & Ji, Y. TCGA-assembler: open-source software for retrieving and processing TCGA data. *Nat Methods* **11**, 599-600 (2014).
35. Rakatomamonjy, A., Bach, F.R., Canu, S. & Grandvalet, Y. SimpleMKL. *J Machine Learning Res* **9**, 2491-2521 (2008).
36. Shen, R., Olshen, A.B. & Ladanyi, M. Integrative clustering of multiple genomic data types using a joint latent variable model with application to breast and lung cancer subtype analysis. *Bioinformatics* **25**, 2906-12 (2009).
37. Mo, Q. *et al.* Pattern discovery and cancer gene identification in integrated cancer genomic data. *Proc Natl Acad Sci U S A* **110**, 4245-50 (2013).
